# Identifying the ideal thermodynamics of non-stoichiometric oxygen-carrier materials for chemical looping water-gas shift†

**DOI:** 10.1039/d4re00454j

**Published:** 2025-01-15

**Authors:** M. Selim Ungut, Ian S. Metcalfe, Wenting Hu

**Affiliations:** a School of Engineering, Newcastle University Newcastle upon Tyne NE1 7RU UK wenting.hu@newcastle.ac.uk

## Abstract

With the growth in importance of H_2_ both industrially and as a potential energy vector along the pathway to achieving future environmental sustainability, there is an increasing need for cleaner and more efficient methods of H_2_ production. One promising short-term solution is to perform the water-gas shift reaction for H_2_ production in a chemical looping reactor to produce separate H_2_ and CO_2_ streams, thereby reducing equipment size and the cost of downstream CO_2_ separation. Non-stoichiometric perovskite oxides have been identified as promising oxygen carrier materials (OCMs) for the chemical looping water-gas shift (CLWGS) process as they can be engineered to allow rapid oxygen uptake kinetics, and also benefit from a thermodynamic advantage allowing higher conversions than that of conventional mixed feed reactor systems at the same temperature. The relationship between the oxygen non-stoichiometry of the solid OCM and the equilibrium oxygen partial pressure of the gas phase streams plays an important role in determining the gas conversions and usable oxygen capacity of the OCM. In this work, an optimal relationship between the two material properties in a thermodynamically limited system is proposed, and an equilibrium packed-bed reactor model is used for validation. The effect on conversions was investigated by varying the thermodynamics of the non-stoichiometric material relative to the proposed optimal case. More generally, an analogue of the pinch analysis can be used to analyse chemical looping water-gas shift reactions and similar processes.

## Abstract 2

As temperature and heat content matching is important in heat exchange, chemical potential and capacity matching is key to chemical looping processes.

## Graphical abstract

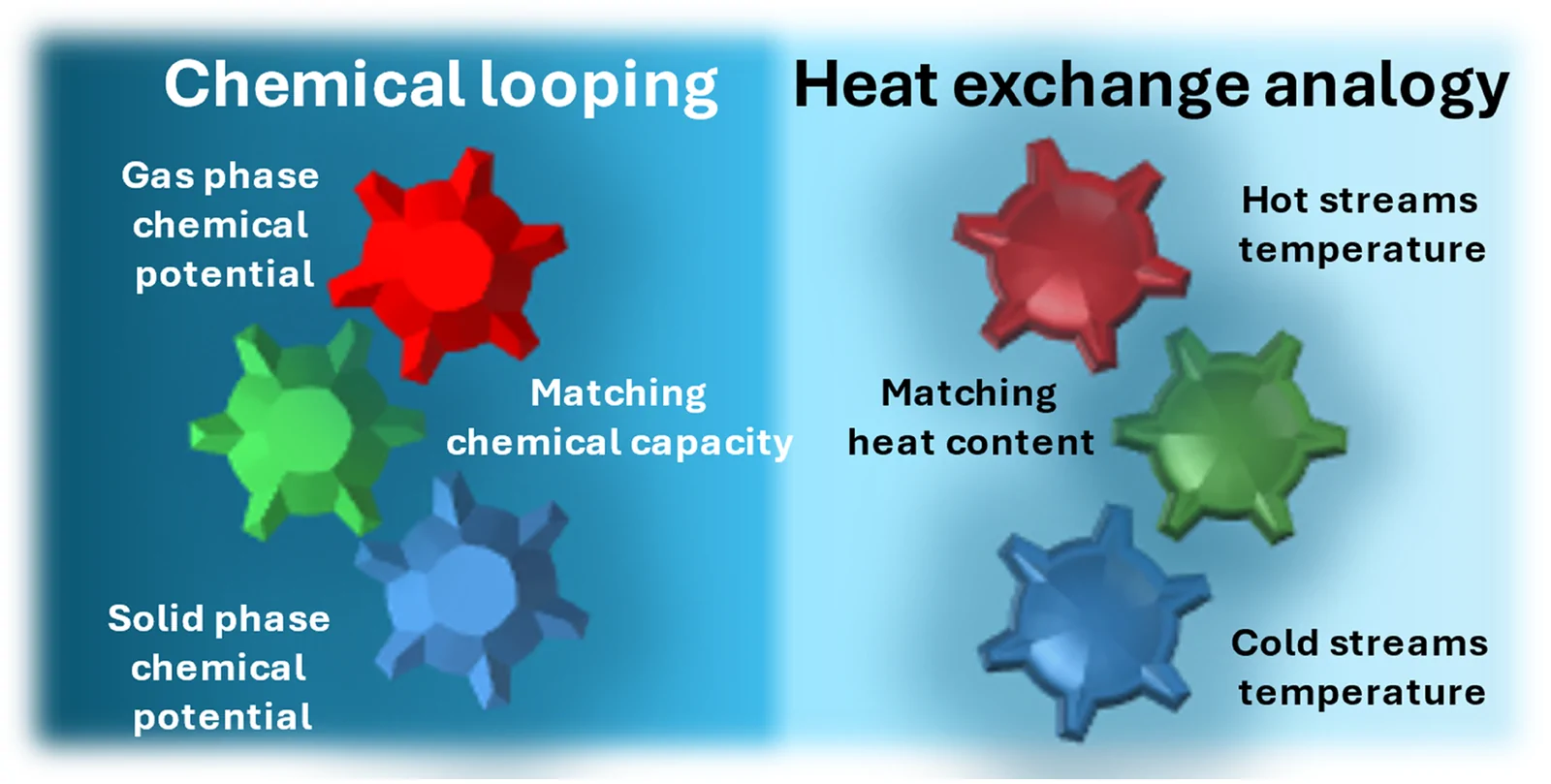

## 1. Introduction

Of the current H_2_ manufacturing processes, the steam reforming process (particularly of natural gas or methane), followed by the water-gas shift (WGS) reaction is the most well-known. About 50% of global H_2_ production is *via* this method and it has a high level of industrial maturity, being in use since the late 1920s.^1,2^ For production of high quality pure H_2_ gas however, the CO_2_ by-product of the WGS reaction must be removed by a separation process, usually pressure swing adsorption, which can add to the final production costs. In addition, the use of multiple reactors at different temperatures requires careful heat integration and the use of specialised catalysts.^3,4^

**Fig. 1:**
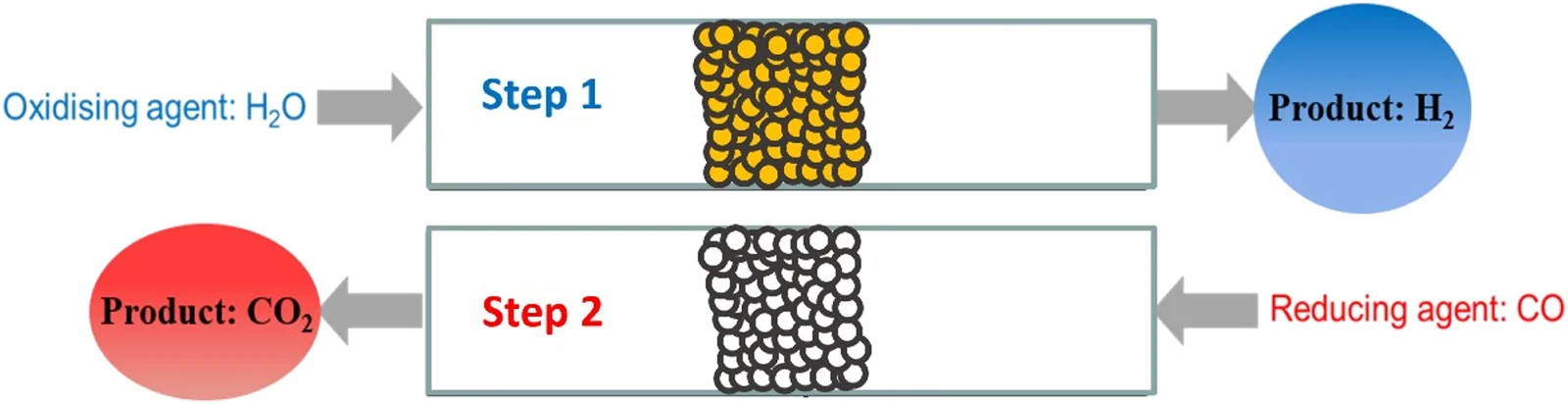
A typical cycle in the CLWGS process using a packed bed reactor with a solid OCM. The bed is first oxidised by flowing through H_2_O, followed by reduction in CO, flowing in the opposite direction.

The chemical looping water-gas shift (CLWGS) process is an alternative technology that can replace the conventional WGS stage of steam methane reforming. In the CLWGS process, temporally separate gaseous H_2_O and CO/syngas feeds are passed over a suitable solid oxygen carrier material (OCM) to produce unmixed H_2_ and CO_2_ product streams^5–9^ as demonstrated for a packed-bed reactor in Fig. 1. The mixed gas-phase WGS reaction (eqn (1)) is split into two separate reactions: an oxidation reaction (eqn (2)) where steam oxidises the OCM resulting in H_2_ being produced, and a reduction reaction (eqn (3)) where CO reduces the OCM thereby producing high purity CO_2_. Here, the OCM is represented as a generic oxide of the form MO_*x*_.

1 Principal reaction: CO + H_2_O ⇌ CO_2_ + H_2_

2 Split reactions with OCM: MO_*x*−*m*_ + *m*H_2_O ⇌ MO_*x*_ + *m*H_2_ (Oxidation of OCM)

3 MO_*x*_ + *m*CO ⇌ MO_*x*−*m*_ + *m*CO_2_ (Reduction of OCM)

The CLWGS process may be incorporated into current industrial plants to achieve inherent CO_2_ separation from the H_2_ stream, and in the short-term be used as a bridge on the path to future low carbon H_2_ production. Depending on the composition of the CO_2_-rich stream produced from eqn (3), further purification of CO_2_ may still be required before transport and storage, but the operation will have been at much smaller scales compared to current practice.

The choice of OCM plays a key role in the performance of CLWGS reactor systems.^10^ Iron oxide has been widely researched for use; it is relatively cheap and has a high oxygen capacity between oxidised and reduced phases.^5,11–13^ However, there are several problems encountered in practice. Iron oxide is a material that exhibits phase changes and oxygen transfer only occurs at fixed chemical potentials. This limits the conversions of the gaseous reactants. For example, the maximum conversion of H_2_O through CLWGS at 1093 K is limited to ∼67% by the metallic iron/wüstite phase transition, and the maximum conversion of CO is limited to ∼67% by the wüstite/magnetite phase transition.^14,15^ In addition, the thermal deactivation of iron oxide due to agglomeration and sintering after only a few redox cycles between haematite and metallic iron states has also been reported,^11^ which leads to poor oxygen exchange kinetics and hence low levels of H_2_ production. This degradation is often mitigated by mixing iron oxide with a support material. Even though the oxygen capacity per mass of the material decreases (typically by a factor of 2–3), high activity can be maintained over time, which is more beneficial to the process.

More recently, research has been carried out into the use of non-stoichiometric perovskite OCMs of the form ABO_3−*δ*_, particularly with the family of strontium doped lanthanum ferrites, La_1−*x*_Sr_*x*_FeO_3−*δ*_ (0 ≤ *x* ≤1).^8,16–18^ In contrast to iron oxide, these OCMs have fast oxygen exchange kinetics between the gas phase and the bulk of the solid phase due to good ionic and electronic conductivities^19–21^ and thus do not require high specific surface area to operate efficiently. In fact, studies have shown that during CLWGS, La_0.7_Sr_0.3_FeO_3−*δ*_ can remain single phase for over 100 cycles under isothermal conditions at 1123 K without any noticeable drop in conversions to H_2_.^18^ This long-term stability of the material is important for industrial scale application.

The fast oxygen transport kinetics of perovskites also leads to the CLWGS process largely being controlled by the gas–solid equilibria of eqn (2) and (3).^22–24^ These equilibria can be quantified by the chemical potential of oxygen, or equivalently, the equilibrium virtual oxygen partial pressure (*p*_O_2__), of the system. In the case of non-stoichiometric perovskites like La_1−*x*_Sr_*x*_FeO_3−*δ*_, the equilibrium *p*_O_2__ varies continuously with the oxygen non-stoichiometry of the solid, *δ*, in addition to the process temperature.^21,25,26^ It has been previously demonstrated that this continuous *δ*–*p*_O_2__ relationship, rather than distinct phase changes at fixed *p*_O_2__, is crucial for the production of high purity H_2_ on a wet basis at high temperatures.^9,23,27^ This needs to be exploited in conjunction with a packed-bed CLWGS reactor or moving bed reactor system where the spatial variation of *p*_O_2__ can be preserved to overcome the chemical equilibrium limitations associated with both the conventional WGS, and a chemical-looping system using stoichiometric OCMs. So far, most of the non-stoichiometric OCMs investigated are transition metal perovskites such as La_1−*x*_Sr_*x*_Fe_1−*y*_Mn_*y*_O_3−*δ*_,^28,29^ La_1−*x*_Sr_*x*_CrO_3−*δ*_,^30^ and La_1−*x*_Sr_*x*_CoO_3−*δ*_;^31^ but non-perovskite metal oxides may also exhibit significant variable non-stoichiometry, *e.g.* cerium-zirconium oxides (Ce_1−*x*_Zr_*x*_O_2−*δ*_).^32^

Another important difference between stoichiometric and non-stoichiometric OCMs is the maximum amount of oxygen available in eqn (2) and (3). For stoichiometric OCMs, the oxygen available is determined by the difference in oxygen stoichiometry between the oxidised and reduced phases. In the case of non-stoichiometric OCMs, this depends on the difference in the degree of oxygen non-stoichiometry at *p*_O_2__ values corresponding to the oxidising and reducing gas streams instead. Furthermore, the purity of gas products depends only on the *p*_O_2__ at which the relevant solid phase transitions occur for stoichiometric OCMs in the thermodynamic limit. Analysis is more complex, and to date unexplored, for the non-stoichiometric case because the *δ*–*p*_O_2__ relationship over the entire range of *p*_O_2__ relevant to the WGS reaction (rather than just the end states) is important.

In this work, the importance of the solid phase *δ*–*p*_O_2__ relationship to the CLWGS process is investigated. An optimal *δ*–*p*_O_2__ relationship for a hypothetical non-stoichiometric OCM is postulated and verified using the 1-dimensional pseudo-homogenous packed-bed reactor model developed by de Leeuwe *et al.*^24^ to assess the impact of the *δ*–*p*_O_2__ relationships of the solid phase on the conversions to H_2_ and CO_2_ products.

## 2. Defining an ideal non-stoichiometric oxygen-carrier material

An ideal non-stoichiometric OCM would exhibit a large change in oxygen content within the range of gas phase *p*_O_2__ relevant to the CLWGS reaction equilibrium where most of the conversion occurs. The virtual *p*_O_2__ of the gas phase during reduction and oxidation steps may be quantified assuming the following gas phase equilibria, respectively,

4 H_2_O ⇌ H_2_ + 1/2O_2_

**Fig. 2:**
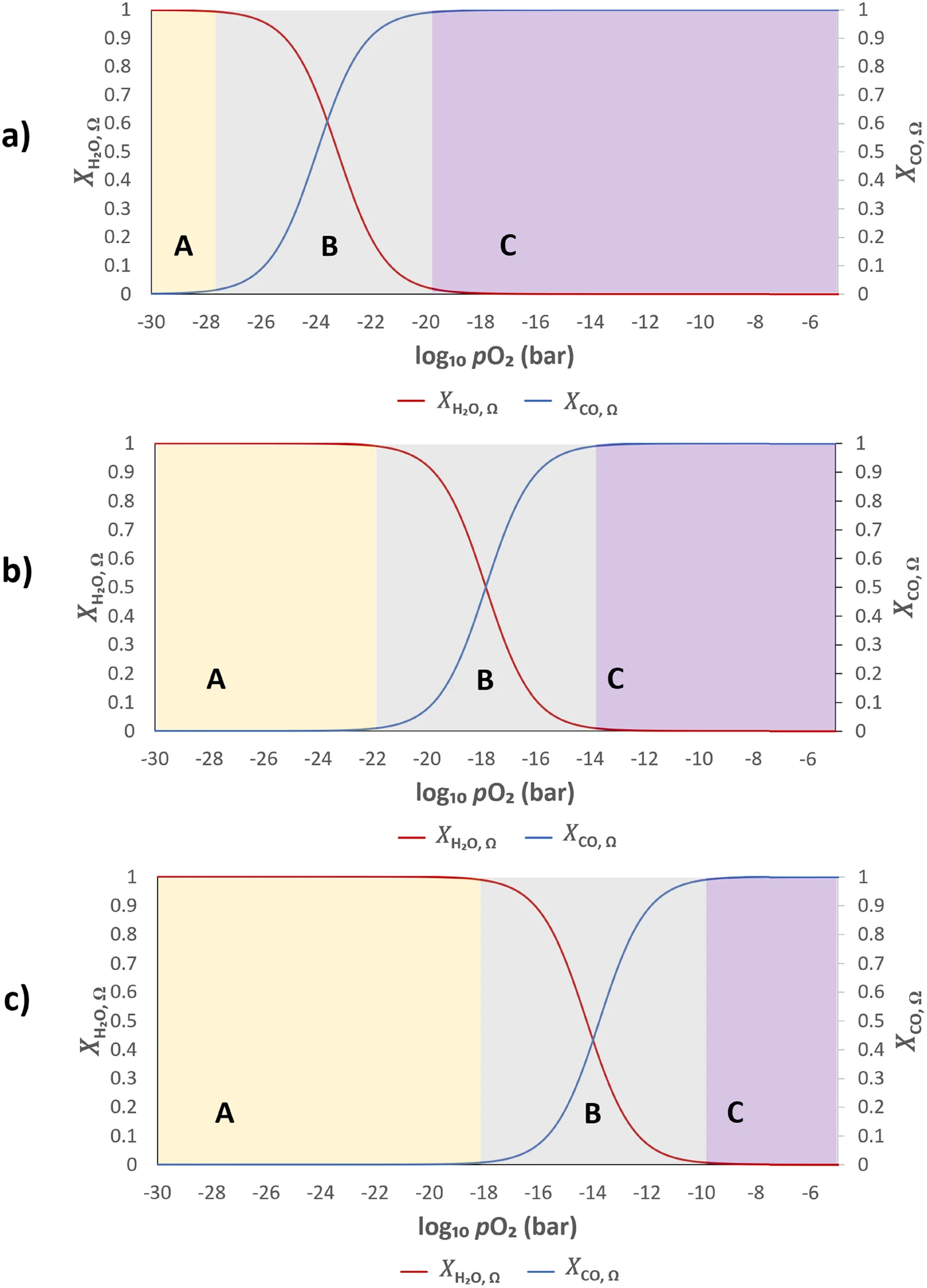
Equilibrium conversions of H_2_O to H_2_, and CO to CO_2_ as a function of log_10_ *p*_O_2__. The effect of changing temperatures at a) 893 K, b) 1093 K and c) 1293 K on the conversions is shown. Each plot is divided into regions labelled A–C, where region B represents the gas phase *p*_O_2__ range most relevant to the reaction equilibrium.

5 CO + 1/2O_2_ ⇌ CO_2_

and can be expressed as6
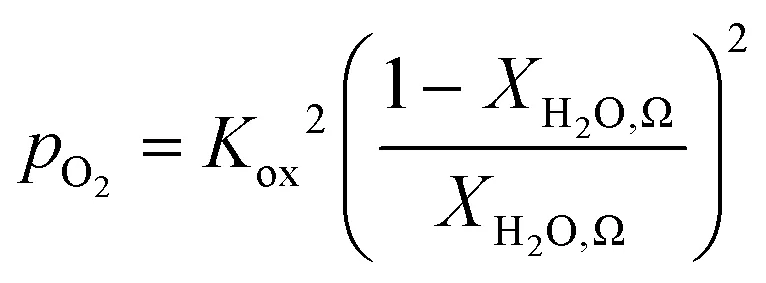
7
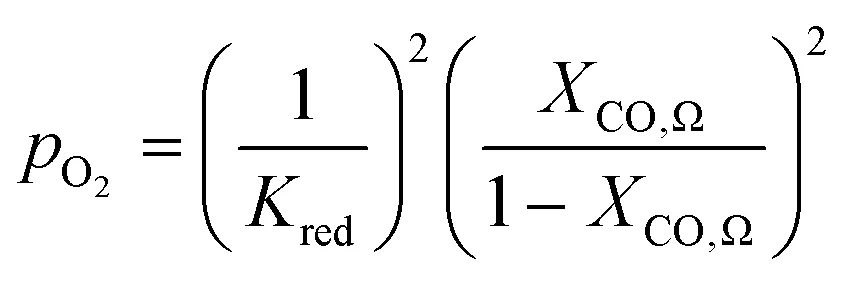
where *K*_ox_ and *K*_red_ are the equilibrium constants of eqn (4) and (5), respectively, and *X*_H_2_O,Ω_ and *X*_CO,Ω_ are the equilibrium conversions of H_2_O and CO. Eqn (6) and (7) are represented graphically in Fig. 2 at several temperatures. The relevant *p*_O_2__ range for CLWGS at 1093 K, for example, is in the region of ∼10^−22^ bar to ∼10^−14^ bar (region B), outside of which one of the equilibrium conversions of the gaseous reactants drops to <1% (regions A and C).

Assuming further that the kinetics of the gas–solid reactions (eqn (2) and (3)) are fast, the gas and solid will rapidly exchange oxygen on contact, and establish a common oxygen potential, or *p*_O_2__. The extent of oxygen exchange is then controlled by the thermodynamics of both the gas and solid. Therefore, it is desirable that most of the available oxygen from the solid resides in a *p*_O_2__ range corresponding to region B in Fig. 2, so that high conversions of both CO and H_2_O can be achieved.

The relative oxygen capacity between the gas phase and solid phase can be represented by a dimensionless number, *λ*_O_, defined as the ratio of usable solid phase oxygen capacity to gas phase oxygen capacity per half cycle in eqn (8),8
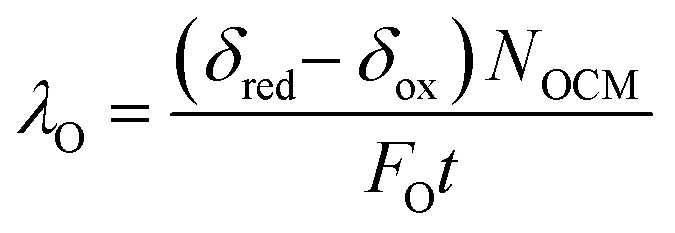
where *δ*_red_ and *δ*_ox_ are the degrees of non-stoichiometry of the OCM in equilibrium with the most reducing and oxidising process gases respectively, *F*_O_ is the molar flow rate of H_2_O or CO (*i.e.* oxygen donor or acceptor) fed into the reactor, *t* is the duration of a half cycle, and *N*_OCM_ is the total number of moles of the OCM present in the packed bed reactor. Further information regarding the properties and implications of *λ*_O_ can be found in the supplementary information.

When *λ*_O_ ≫ 1, the gas phase is the limiting reactant, and the conversion of CO or H_2_O will be largely limited by chemical equilibrium; when *λ*_O_ ≪ 1, the solid phase is the limiting reactant, and the conversion of the gas will be limited by the oxygen capacity of the solid. Here, the optimal *δ*–*p*_O_2__ relationship for a non-stoichiometric OCM for CLWGS at any given temperature is defined to be one that gives the highest possible conversion at *λ*_O_ = 1. It is postulated (and verified later in this work) that such a relationship would have a shape matching as closely as possible that of the gas-phase equilibria plotted in Fig. 2 (when scaled by the amount of oxygen available, *δ*_max_–*δ*_min_) in the range where the change in conversion is significant, and centred around the *p*_O_2__ where *X*_H_2_O,Ω_ = *X*_CO,Ω_. The mathematical representation of the optimal cases is presented as follows.

At 1093 K, the WGS equilibrium is close to unity, therefore9
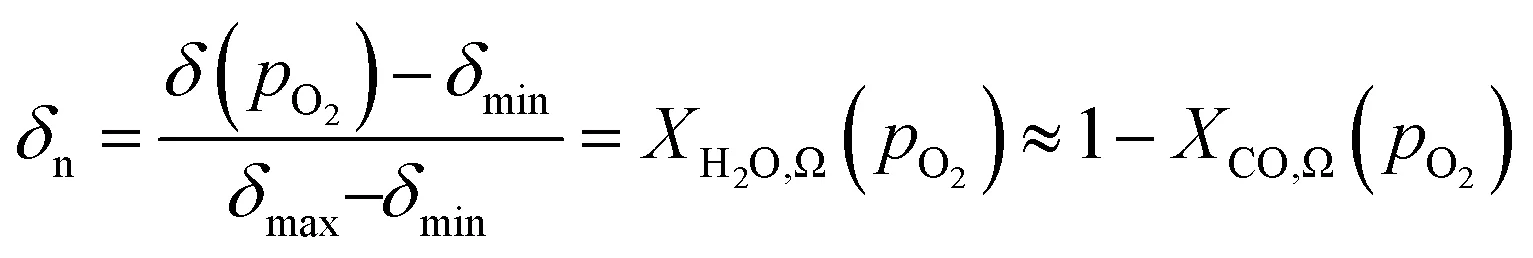
where *δ*_n_ is the normalised degree of oxygen non-stoichiometry of the OCM. *δ*_max_ and *δ*_min_ represent the maximum and minimum oxygen non-stoichiometry achievable. The equilibrium conversions of gases are related to *p*_O_2__ by eqn (6) and (7), respectively.

The solid phase *δ*–*p*_O_2__ relationship can be closely approximated to the shapes of the gas phase conversion curves using a logistic function, with two adjustable parameters, as described in eqn (10).10
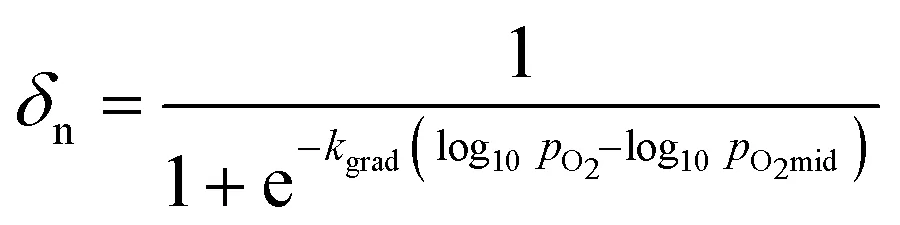
Here, *p*_O_2_mid_ is the *p*_O_2__ value of the midpoint of the sigmoid (10^–17.8^ bar at 1093 K) and *k*_grad_ is the steepness. The value of *k*_grad_ (−1.15 at 1093 K) can be determined by minimising the difference between eqn (10) and (9). The logistic function is chosen due to its simplicity compared to other commonly used sigmoid functions.

At temperatures other than 1093 K, *X*_H_2_O,Ω_(*p*_O_2__) ≠ 1 − *X*_CO,Ω_(*p*_O_2__), and the consequence of this will be examined further in the results section. In short, the optimal *δ*(*p*_O_2__) in this case should be such that *δ*_n_ is equidistant from *X*_H_2_O,Ω_(*p*_O_2__) and 1 − *X*_CO,Ω_(*p*_O_2__).

## 3. CLWGS reactor model

An ideal packed-bed reactor model is used to evaluate the influence of the thermodynamics of the non-stoichiometric OCM on the gas phase conversion of CLWGS. The model used here follows that described by de Leeuwe *et al.*^24^ To summarise, a pseudo-homogeneous model is used to represent a packed-bed reactor under isothermal conditions, and without mass transport limitations. The effects of axial dispersion and radial gradients are ignored as they provided minimal improvement to results and do not justify the ∼20 times increase in computation time.^24^ It is assumed that the kinetics of the gas–solid reactions are very fast, and the reactions are thermodynamically limited; and possible side reactions such as the reverse Boudouard reaction do not occur.

The packed-bed reactor is cyclically fed with an equimolar flow of CO or H_2_O in reverse flow configuration for the reduction and oxidation of the OCM, respectively. The mass balance of the system is described by the following system of equations,11
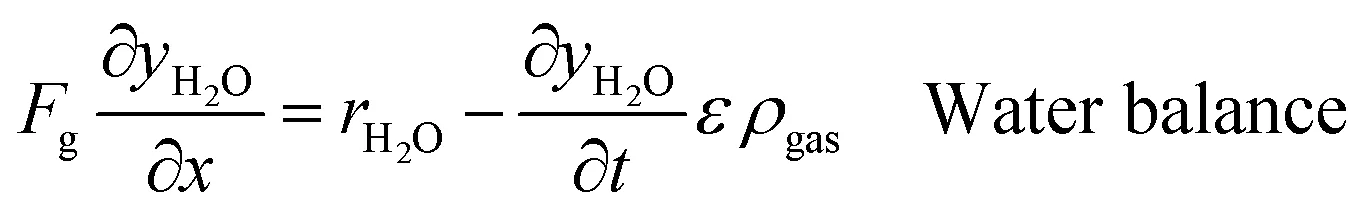
12
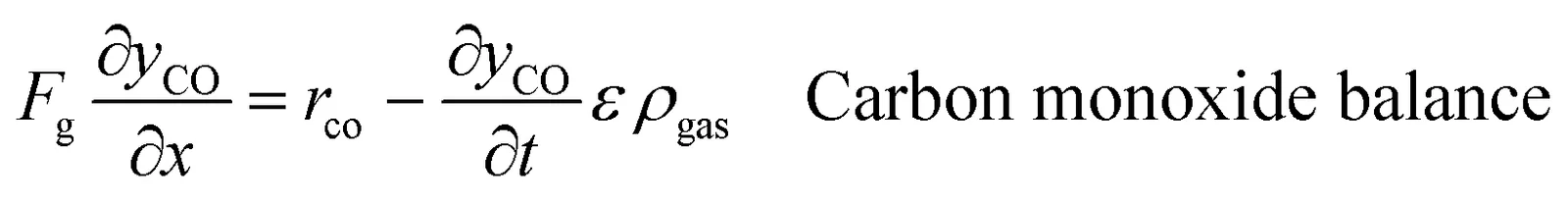
13
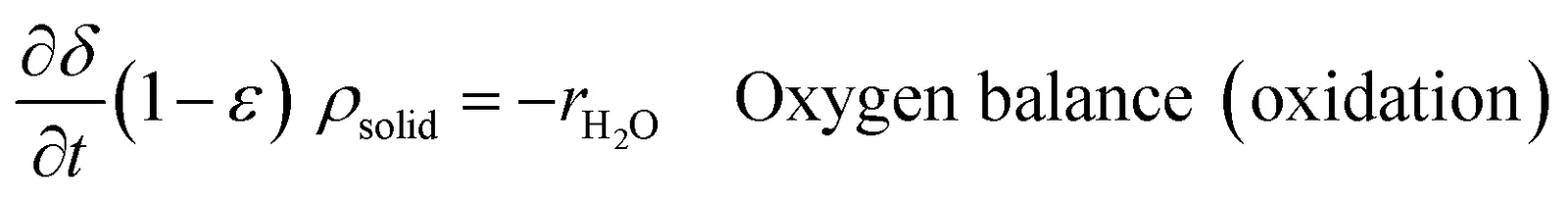
14
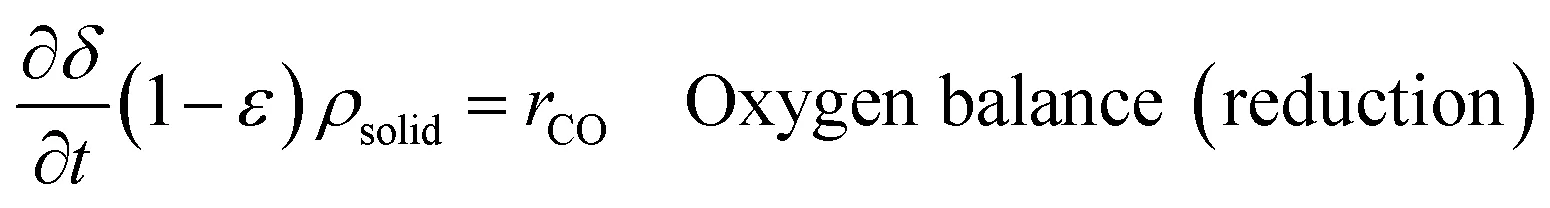

15 *y*_H_2__ + *y*_H_2_O_ = *y*_H_constant__ Hydrogen balance

16 *y*_CO_ + *y*_CO_2__ = *y*_C_constant__ Carbon balance

where *F*_g_ is the molar flowrate of the gas stream; *ρ*_gas_ and *ρ*_solid_ represent the molar density of the gas phase and the OCM, respectively; *x* represents axial position along the length of the bed; *ε* is the voidage of the bed; the rate of reaction of H_2_O and CO and, *r*_H_2_O_ and *r*_CO_ are linked to the rate of change of oxygen content of the solid OCM at any given location, 
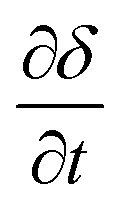
, *via*eqn (11) and (12), respectively; *y*_*i*_ represents the mole fraction of species *i* in the gas phase. The total mole fraction of hydrogen containing species (*y*_H_constant__) and that of the carbon containing species (*y*_C_constant__) are invariant in the system due to the assumption of no axial dispersion and that there is no carbon deposition. For the purposes of this work, both are set to 5%, corresponding to the experimental conditions used in de Leeuwe *et al.*^24^

Furthermore, for an equilibrium limited process at constant temperature and pressure, the change in solid oxygen content can be linked to the change in the gas phase mole fractions.17
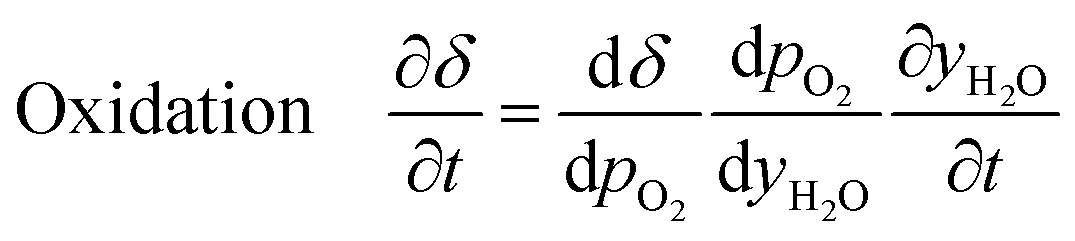
18
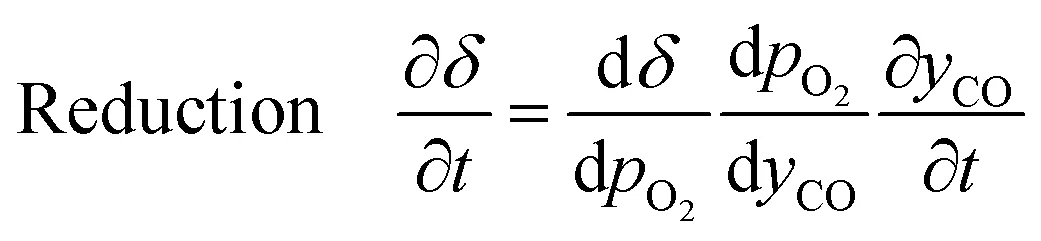
The terms 
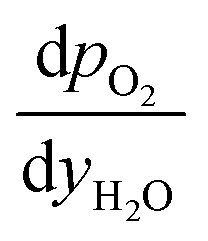
 and 
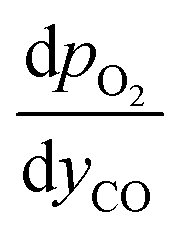
 are derived from eqn (6) and (7), and are given as follows.19
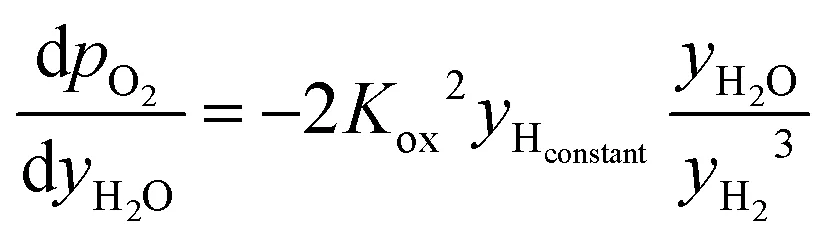
20
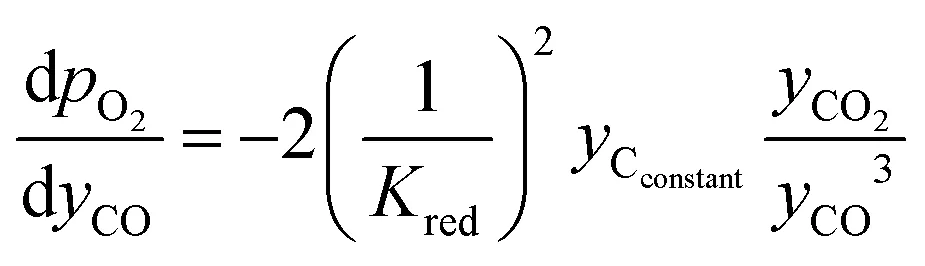
Eqn (11) and (12) were transformed into a system of time-dependent ODEs using the upwind scheme to discretise the spatial variable, and the ode45 solver in MATLAB was used to compute solutions with a total of 100 grid points. A numerical experiment was carried out using double and half the number of grid points to verify that the solutions were independent of grid spacing.

The *p*_O_2__ of the inlet streams are ill defined due to the lack of any H_2_ or CO_2_ in the normal composition of the inlet gases. Instead, the *p*_O_2__ of these streams are approximated as the equilibrium *p*_O_2__ of water splitting and CO disproportionation at the reaction temperature, respectively. This results in a very small amount of H_2_ and CO_2_ being present in the inlet streams. This will not influence the mass balance appreciably, whilst provide a well-defined boundary condition for the problem (*p*_O_2__ ∼ 10^−8^ bar for the H_2_O stream and ∼10^−23^ bar for the CO stream at 1073 K). For the initial condition used for each half cycle, the *p*_O_2__ values across the packed bed at the start of the given half cycle (*e.g.* reduction) were set to be the same as those from the end of the previous half cycle (oxidation) by using eqn (6) or (7). These approximations do not change the oxygen balance of the system appreciably due to the system operating at very low *p*_O_2__ values.^23,24^ For the first half cycle, an arbitrary initial condition (*p*_O_2__ of solids = *p*_O_2__ of the CO inlet stream) was used. This choice is inconsequential here since only the results at cyclic steady state are of interest.

## 4. Results

### Validation of optimal *δ*–*p*_O_2__ relationship at 1093 K

As an initial validation, the reactor model was run using both a *δ*–*p*_O_2__ relationship according to eqn (9), which is linked to the analytical equilibrium gas phase expressions (given in eqn (6) and (7)), and the approximation using the logistic function (eqn (10)). Identical results were obtained within at least 3 significant figures, so further manipulation of the relationship was performed through the logistic function only.

**Fig. 3:**
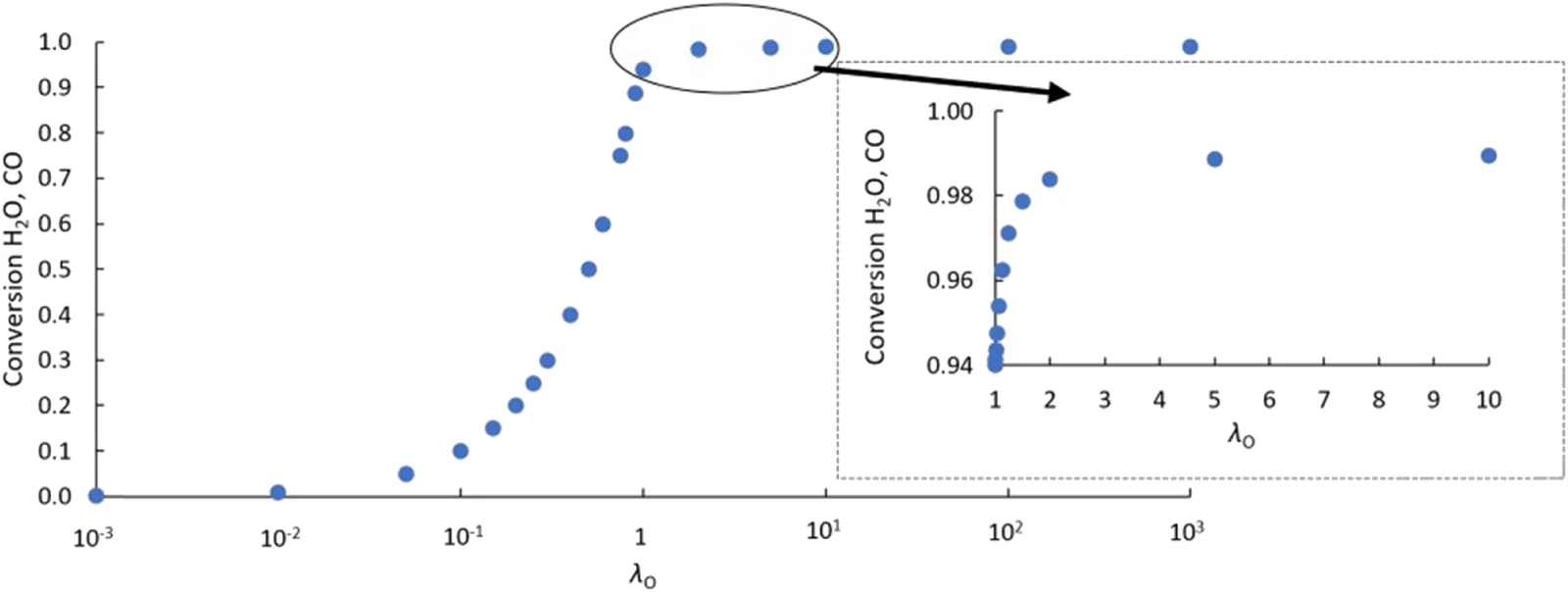
Values of *X*_H_2_O_ and *X*_CO_ achieved at different values of *λ*_O_ at operating temperature of 1093 K using the CLWGS reactor model. The data is taken during steady cycling where the difference between the values of *X*_CO_ and *X*_H_2_O_ in successive half cycles was <0.01. The inset shows more data points in the range 1 ≤ *λ*_O_ ≤ 10.

The results in Fig. 3 show that with the postulated optimal *δ*–*p*_O_2__ relationship, *X*_CO_ and *X*_H_2_O_ approach 1 asymptotically as *λ*_O_ → ∞. The conversion when *λ*_O_ = 1 is ∼94%. It increases by ∼4.5% when increasing the value of *λ*_O_ from 1 to 2, and by a further ∼0.4% when increasing *λ*_O_ from 2 to 10. Therefore, it is not worth increasing *λ*_O_ much further beyond a value of ∼2 because the increase in product purity is too low.

**Fig. 4:**
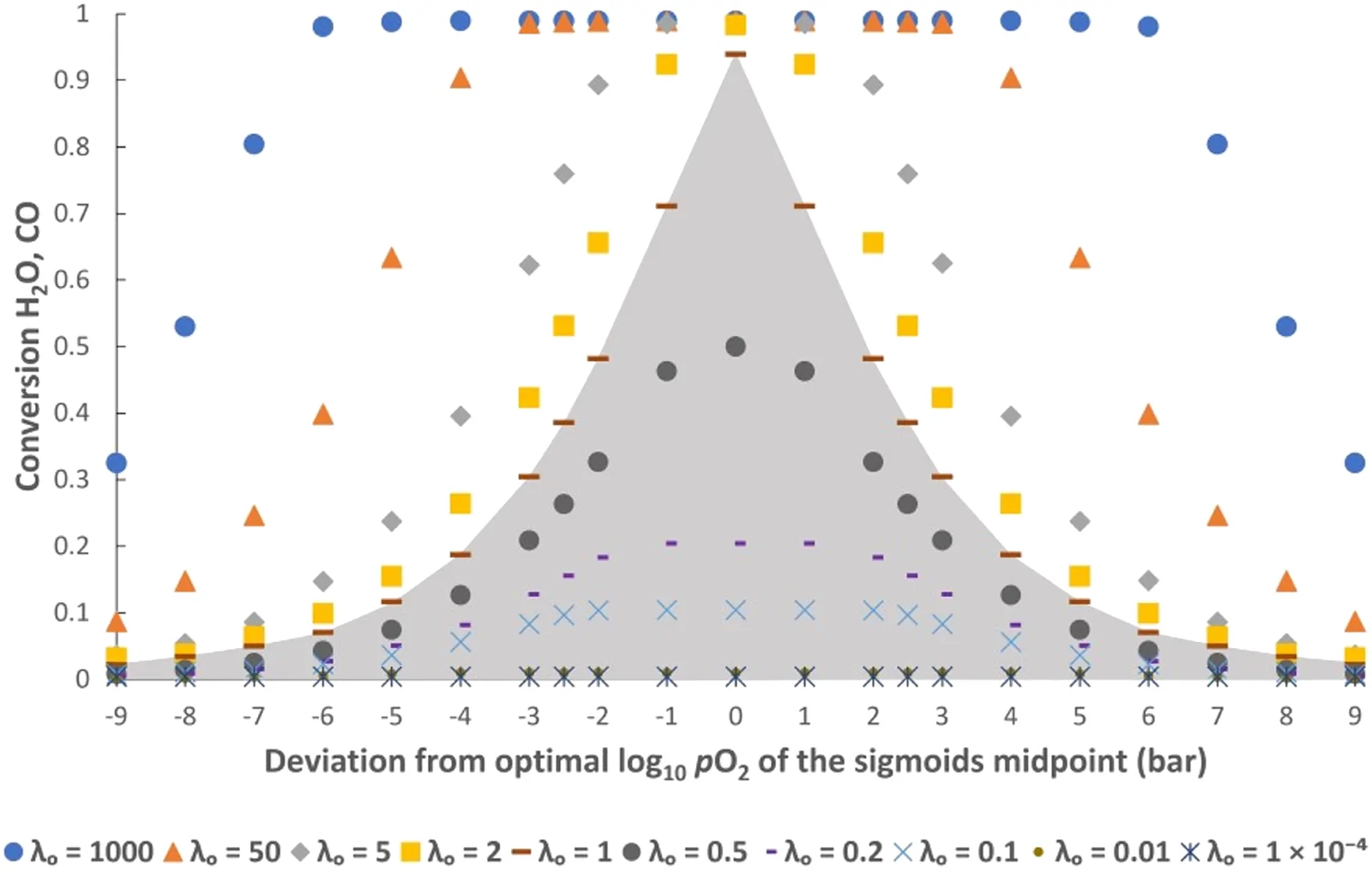
Steady state conversions determined from the simulation using different values for log_10_ *p*_O_2_mid_ at various values of *λ*_O_. Midpoint values are given in reference to the deviation from the optimal midpoint of log_10_ *p*_O_2_mid_ = −17.8. The value of *k*_grad_ is kept constant at −1.15. The grey shaded area indicates regions where the solid phase is the limiting reactant.

**Fig. 5:**
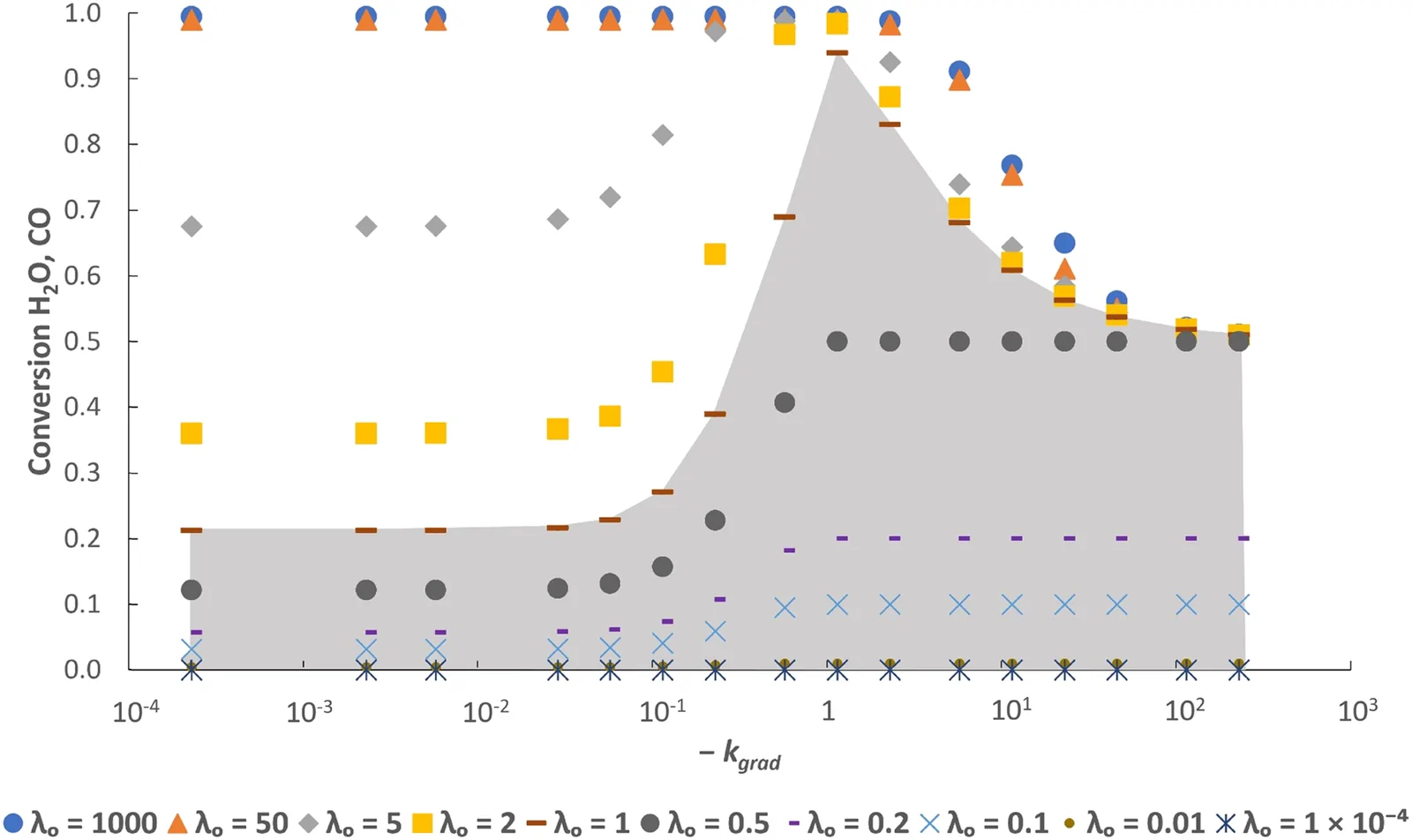
Steady state conversions determined from the simulation using different values for *k*_grad_ at various values of *λ*_O_*.* The value of log_10_ *p*_O_2_mid_ is kept constant at −17.8. The grey shaded area indicates regions where the solid phase is the limiting reactant.

It is worth noting that the values of *X*_CO_ and *X*_H_2_O_ are only dependent on the values of log_10_ *p*_O_2_mid_, *k*_grad_ and *λ*_O_ used in eqn (9). Any combination of *δ*_red_–*δ*_ox_, *F*_O_, *t* and *N*_OCM_ can be used to give the same value of *λ*_O_ without altering *X*_CO_ and *X*_H_2_O_. Importantly, any deviation from the optimal values of log_10_ *p*_O_2_mid_ and *k*_grad_ (*i.e.* those leading to eqn (9) closely matching eqn (6) and (7)) leads to a decrease in *X*_CO_ and *X*_H_2_O_ at cyclic steady state, thus validating the postulation posed. Fig. 4 shows this for different values of log_10_ *p*_O_2_mid_ and Fig. 5 shows this for different values of *k*_grad_.

In addition, it is also clear from the figures that when the gas phase is the limiting reactant (*i.e. λ*_O_ ≥ 1), it is possible to have a less-than-optimal value of log_10_ *p*_O_2_mid_ or *k*_grad_ while still achieving high conversions. For example, at *λ*_O_ ≥ 50 the decrease in conversions when deviating from the optimal log_10_ *p*_O_2_mid_ by ± 3, or deviating from the optimal *k*_grad_ value by a decrease of ∼2 orders of magnitude or an increase by a factor of 2, is fairly small (∼1%). However, this means that more solids will be required than in the optimal case to achieve the same production rate of gaseous products, and this will negatively impact on the capital cost and/or operating cost of a potential process.

Furthermore, Fig. 5 shows that a broader or steeper *δ*–*p*_O_2__ relationship results in less of the oxygen capacity of the OCM being usable during reaction, and hence gives lower conversions despite the total oxygen capacity, *λ*_O_, being unchanged. At very low |*k*_grad_| values, the *δ*–*p*_O_2__ relationship approaches a straight line on the semi-log scale, and the conversion becomes independent of *k*_grad_. Despite a poorer *p*_O_2__ distribution, a very large *λ*_O_ value results in only a marginally lower value of conversion. On the other hand, at very high values of |*k*_grad_| the *δ*–*p*_O_2__ relationship approaches that of an OCM with a single phase transition at *p*_O_2__ = 10^−17.8^ bar and therefore the conversion tends towards 50% as expected.^23^

### Optimisation at temperatures other than 1093 K

**Fig. 6:**
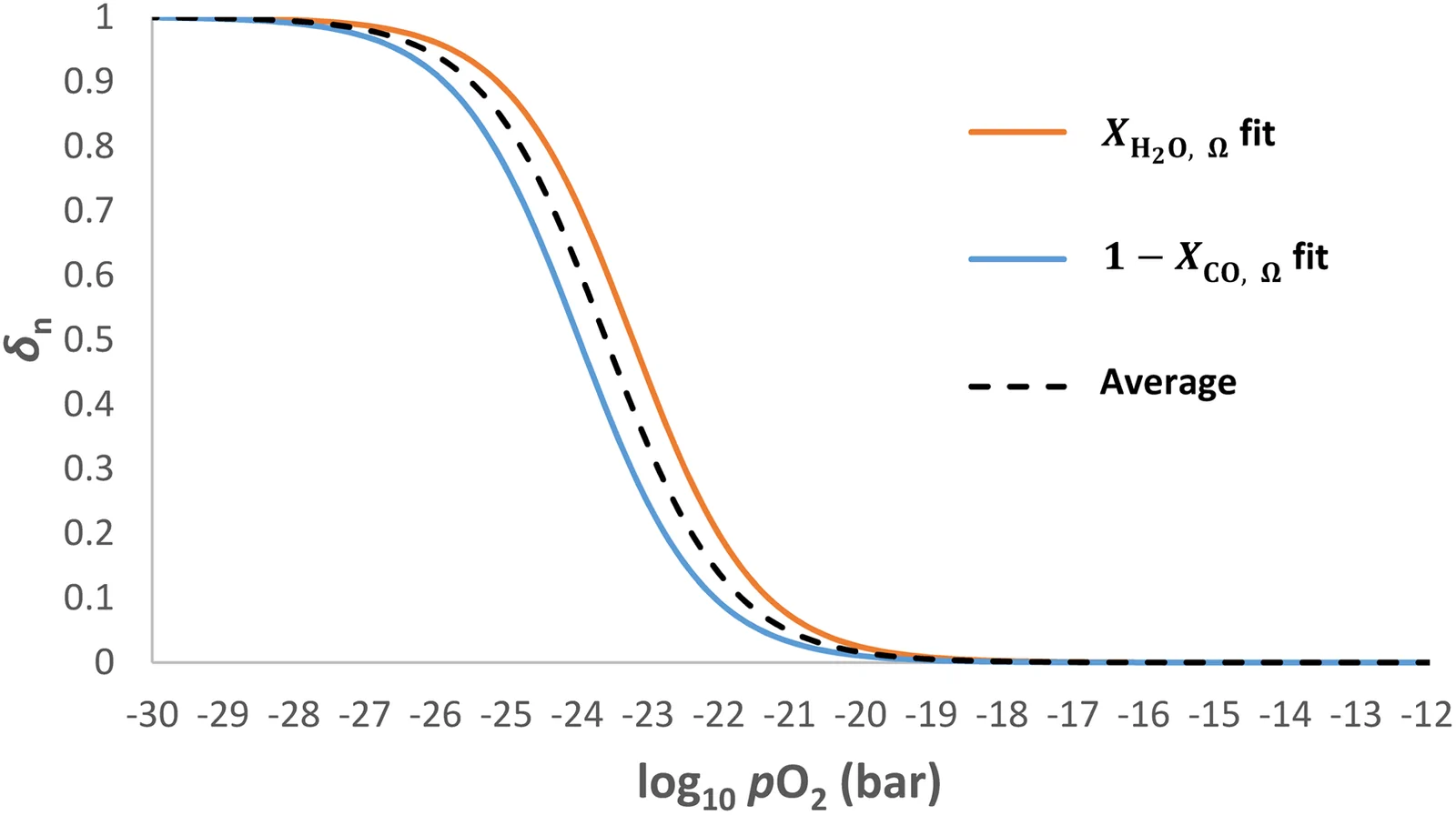
Example *δ*_n_*vs.* log_10_ *p*_O_2__ profiles at *T* = 893 K. The log_10_ *p*_O_2_mid_ value of the case “average” is found by taking the average of the log_10_ *p*_O_2_mid_ values for the “*X*_H_2_O,Ω_ fit” and “1 − *X*_CO,Ω_ fit” cases that represent the oxidation and reduction half cycles respectively.

At temperatures other than 1093 K, the Gibbs free energy of reaction for WGS is non-zero. Therefore 
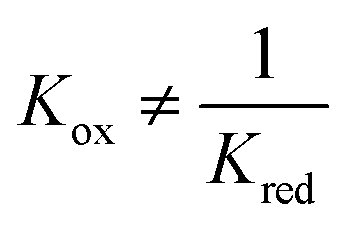
 and the gas conversion-*p*_O_2__ relationships (eqn (6) and (7)), as shown in Fig. 2, translate along the abscissa by different amounts whilst maintaining the same shape, hence sharing a common *k*_grad_ but different log_10_ *p*_O_2_mid_. In such cases, the optimal log_10_ *p*_O_2_mid_ value for eqn (9) is found to be the average of the log_10_ *p*_O_2_mid_ values fitted to the curves for *X*_H_2_O,Ω_ and *X*_CO,Ω_, respectively, as illustrated in Fig. 6. This is demonstrated in Table 1 (scenario B) and compared to the case when log_10_ *p*_O_2_mid_ matches the gas phase equilibrium of either eqn (4) or (5) (scenario A).

**Table 1:** Simulated conversions at different temperatures for *λ*_O_ values of 0.5, 1 and 2. At each temperature the simulation is run for two scenarios: A) log_10_ *p*_O_2_mid_ is chosen to match the gas phase equilibrium of eqn (4) (matching eqn (5) instead would yield identical results), and B) log_10_ *p*_O_2_mid_ is set to the average of the log_10_ *p*_O_2_mid_ values matching eqn (4) and (5), respectively. Conversions are shown for optimal *k*_grad_, as well as scenarios where the gradient is halved or doubled

Reaction temperature (K)	Choice of log_10_ *p*_O_2_mid_	Conversion when *k*_grad_ is half the optimal value			Conversion when *k*_grad_ is optimal			Conversion when *k*_grad_ is twice the optimal value			Equilibrium conversion of homogeneous WGS
		*λ*_O_ = 0.5	*λ*_O_ = 1	*λ*_O_ = 2	*λ*_O_ = 0.5	*λ*_O_ = 1	*λ*_O_ = 2	*λ*_O_ = 0.5	*λ*_O_ = 1	*λ*_O_ = 2	
**593**	Scenario A	0.45	0.84	1.00	0.50	0.96	1.00	0.50	0.84	0.91	0.85
	Scenario B	0.48	0.92	1.00	0.50	1.00	1.00	0.50	0.99	0.99	
**893**	Scenario A	0.43	0.76	1.00	0.50	0.96	1.00	0.50	0.84	0.91	0.61
	Scenario B	0.43	0.77	1.00	0.50	0.99	1.00	0.50	0.91	0.94	
**993**	Scenario A	0.42	0.72	1.00	0.50	0.96	1.00	0.50	0.84	0.90	0.55
	Scenario B	0.42	0.73	1.00	0.50	0.98	1.00	0.50	0.87	0.91	
**1193**	Scenario A	0.40	0.66	0.90	0.50	0.88	0.92	0.50	0.78	0.84	0.46
	Scenario B	0.40	0.66	0.90	0.50	0.89	0.92	0.50	0.80	0.84	
**1293**	Scenario A	0.39	0.63	0.84	0.50	0.83	0.86	0.50	0.72	0.80	0.43
	Scenario B	0.39	0.63	0.85	0.50	0.84	0.86	0.50	0.76	0.81	

Table 1 shows that scenario B indeed gives better conversions for CLWGS compared to the corresponding scenario A, although the difference is only marginal in most cases. This improvement in conversion is more noticeable at the lower temperatures (*i.e.* at 593 K), as the difference in log_10_ *p*_O_2_mid_ between the two scenarios is larger. Furthermore, the improvement in conversion is most noticeable when *λ*_O_ is close to 1, where the conversion is not dominated by the imbalance of oxygen capacity between the gas phase and solid phase. It is also clear from Table 1 that the conversions to products become more favourable at lower temperatures. However, the effect of kinetic limitations will become more severe, which has not been considered here.

From a thermodynamic point of view, the optimal case needs to satisfy
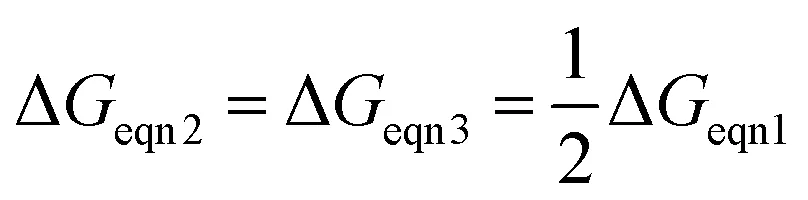
at the reaction temperature over the full range of *p*_O_2__ where the reactions occur, which can be rather restrictive. However, it is evident from results presented in Table 1 that moderate deviations from this constraint are not detrimental to the effectiveness of the chemical looping scheme. In comparison to deviations in log_10_ *p*_O_2_mid_, it is more important to ensure that Δ*G*_eqn2_ and Δ*G*_eqn3_ are close to constant (which is much easier to satisfy) so that *k*_grad_ is close to the optimal value.

## 5. Discussions

### Impact of parameters contributing to *λ*_O_ on process economics

The individual parameters contributing to *λ*_O_ do not influence the thermodynamic efficiency of the chemical looping water-gas shift process if *λ*_O_ remains unchanged, as shown in the supplementary information. However, it is clear that these parameters will influence the size of the reactor needed for a given throughput of gas products – all else being equal, oxygen carrier materials with a larger oxygen transfer capacity per volume will be preferred since the associated reactor footprint will be smaller.

Non-stoichiometric mixed metal oxides usually have much lower oxygen transfer capacity per volume compared to simple, stoichiometric metal oxides such as iron oxides. Although the cycle time can be shortened to compensate for cases where the oxygen transfer capacity is low but the *δ*–*p*_O_2__ relationship is more favourable, there will be operational limits on the frequency of gas switching. For instance, there is a theoretical lower limit to the gas switching frequency, corresponding to when the half-cycle duration is comparable to the residence time of gas within the reactor. More frequent gas switching will also likely be linked to higher operation and maintenance costs. Therefore, although beyond the scope of the current work, there will be further trade offs between thermodynamic efficiency and the economic efficiency of the processes that need to be considered.

### Analogy to heat exchange and pinch analysis

**Fig. 7:**
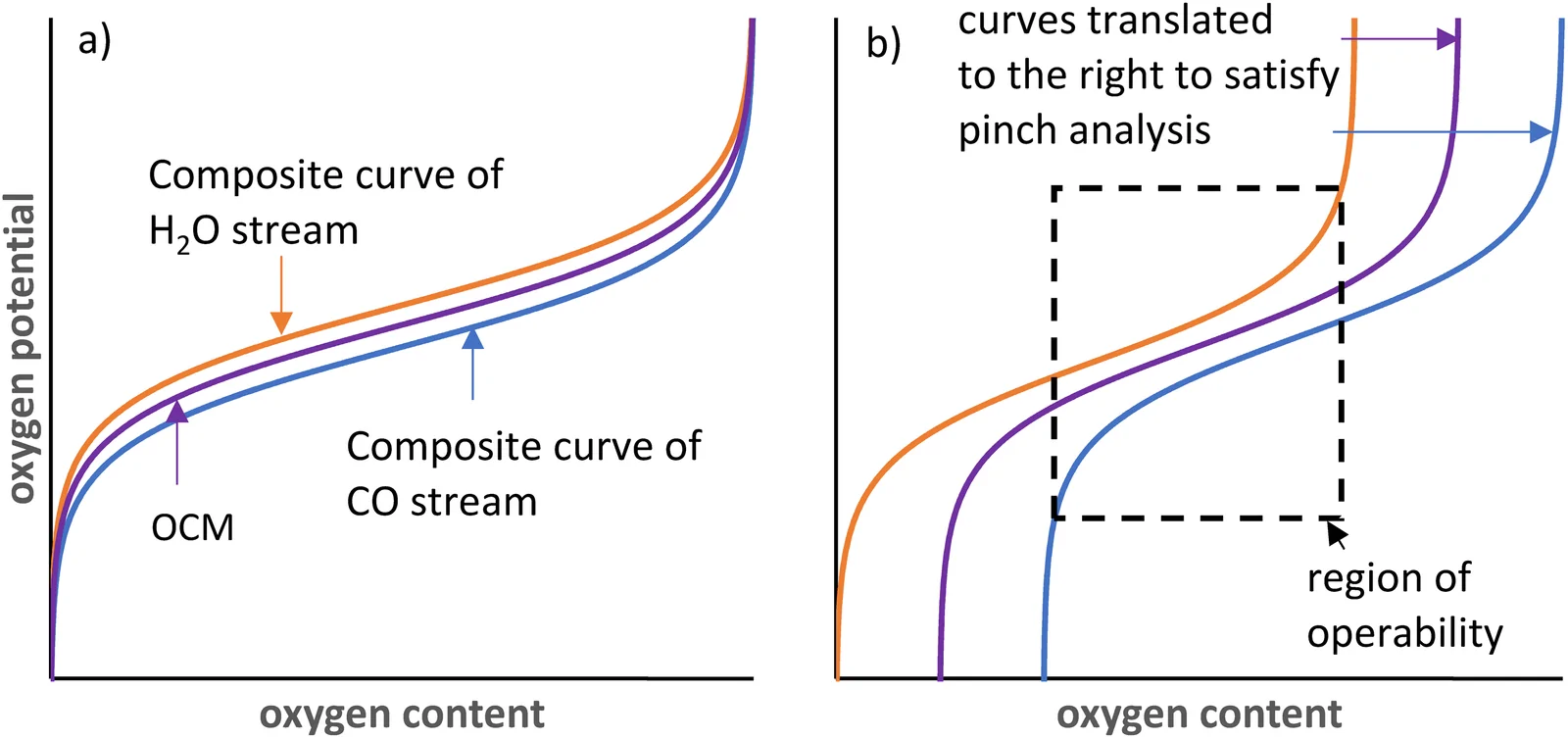
Illustration of the pinch analysis for CLWGS based on an analogy to heat exchangers and composite curves. a) for cases below 1093 K and b) for cases above 1093 K. The composite curve for the H_2_O stream is shown in orange, the OCM in purple and the CO stream in blue. In a) the full range of oxygen content can be utilised whereas in b), only the section within the dashed black box can be used.

The oxygen exchange between the gas phase and solid phase in CLWGS processes is analogous to the heat exchange between two streams in a heat exchanger.^33^ In such an “oxygen exchanger”, the oxygen chemical potential corresponds to the temperature of each material stream, and the oxygen content available for exchange corresponds to the heat content of each stream, as shown in Fig. 7. This can be further generalised to any chemical reaction with two unmixed material streams (for example, either as different phases, or separated by a membrane). Indeed, this has been recently utilised to rationalise efficient chemical-looping reverse water-gas shift and methane reforming.^27,34^

The equivalent of pinch analysis for heat exchangers can be used to further validate the optimality of the solid *δ*–*p*_O_2__ relationship relative to the gas phase thermodynamics identified in this work. It is obvious from analysis of heat exchangers that counter-current operation is more efficient than co-current operation (or a well-mixed system). Therefore, only the counter-current case is considered here.

The CLWGS process typically involves two “oxygen exchangers” operating in sequence. For each “oxygen exchanger”, the most efficient counter-current operation configuration is for the shape of the “composite curves” (*i.e.* the oxygen potential–oxygen content relationship) to match exactly. It may be necessary to have a small, constant offset in oxygen chemical potential if exchange kinetics become rate limiting, but the wider this gap is, the greater the exergy loss that is incurred. In particular, if the shapes of the composite curves are different, then a pinch point will exist and all other points on the composite curves will diverge from each other, leading to larger inefficiencies. This is reflected in the lower gas conversions achieved in suboptimal cases presented in Table 1. Furthermore, when the oxygen contents of the two streams are also matched (*λ*_O_ = 1), the full oxygen content of both material streams can be used, without the need of any “utility streams”, resulting in an efficient system. This leads to the minimum size of the reactor and amount of OCM being used for the reaction.

In the case of CLWGS, H_2_O is the oxygen rich stream, therefore represented by the “hot” composite curve; and the OCM is represented by the “cold” composite curve. Conversely, for the reaction of CO with the OCM, the OCM is more oxygen rich and is represented by the “hot” composite curve. Consequently, for a feasible system it necessitates that the order of *p*_O_2__ of each of the material streams at any “oxygen content” shown in Fig. 7 must satisfy *p*_H_2_O stream_ ≥ *p*_OCM_ ≥ *p*_CO stream_.

At 1093 K, 
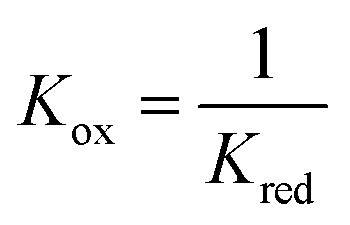
, therefore *p*_H_2_O stream_ = *p*_CO stream_ and must also be equal to *p*_OCM_. At temperatures lower than 1093 K, *p*_H_2_O stream_ > p_CO stream_ (Fig. 7a), and *p*_OCM_ should be such that the OCM's corresponding composite curve lies halfway between the composite curves of the H_2_O and CO streams. In this way, the efficiencies of both gas–solid reactions are comparable, otherwise the conversion of the overall process will be limited to the less efficient gas–solid reaction.^23,33^ At temperatures greater than 1093 K, *p*_H_2_O stream_ < *p*_CO stream_ for a given oxygen content. In this case, to satisfy the pinch analysis, the cold composite curve will have to translate to the right until the curve is partially underneath the hot composite curve before oxygen exchange can take place (the purple and blue lines in Fig. 7b). Consequently, this leads to a lower amount of oxygen content being transferred between streams, and the gas conversions achievable are also lower (limited to the region within the dashed box in Fig. 7b).

### Approaching the ideal thermodynamics in practice

**Fig. 8:**
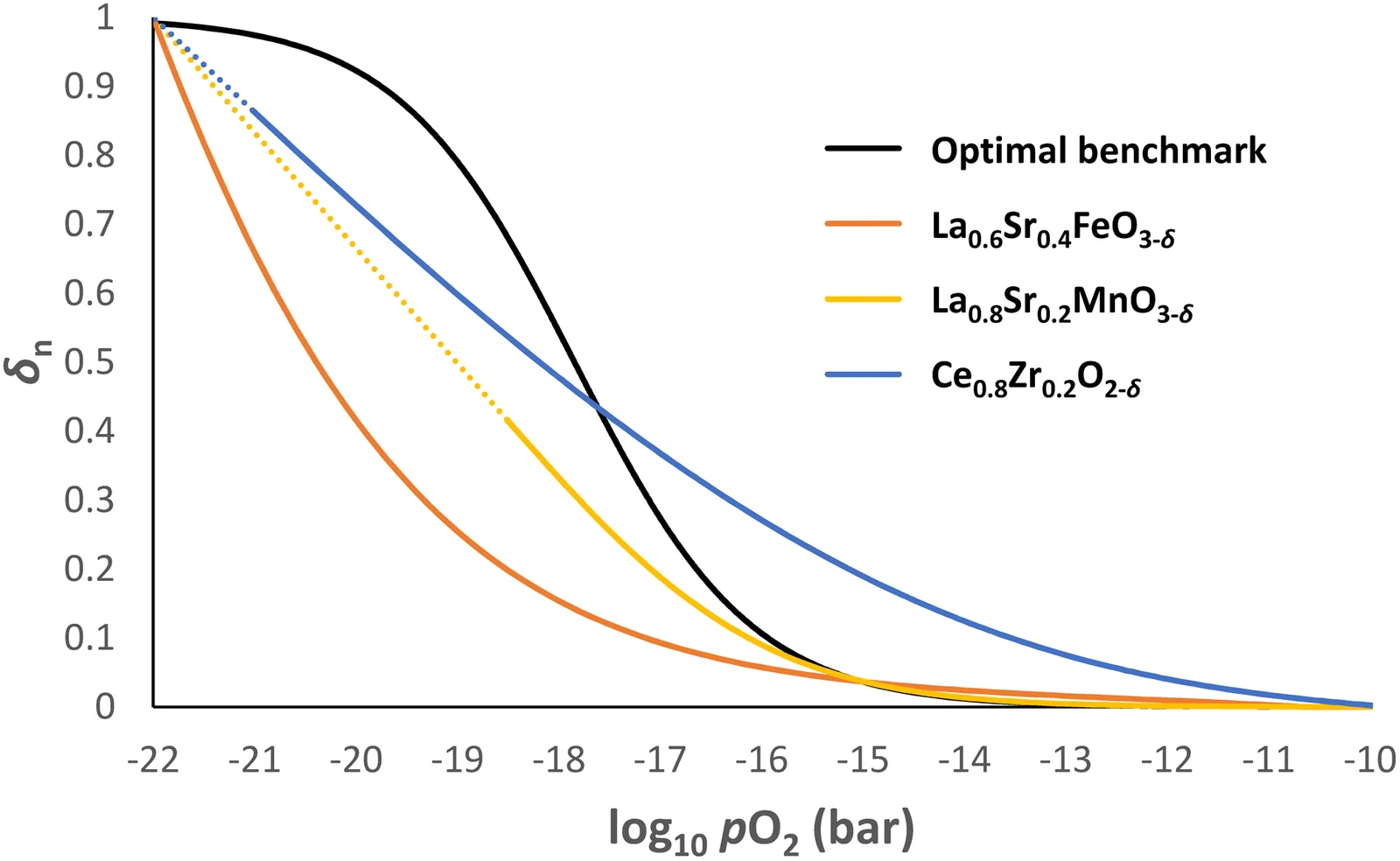
*δ*_n_ *vs.* log_10_ *p*_O_2__ profiles for La_0.6_Sr_0.4_FeO_3−*δ*_, La_0.8_Sr_0.2_MnO_3−*δ*_ and Ce_0.8_Zr_0.2_O_2-*δ*_ at 1093 K using data from literature.^26,29,32^ The optimal *δ*–*p*_O_2__ relationship is also given as a benchmark. The dotted lines for La_0.8_Sr_0.2_MnO_3−*δ*_ and Ce_0.8_Zr_0.2_O_2−*δ*_ indicate extrapolation of the data if no decomposition were to occur.

In practice, it is almost impossible to design a single OCM that possesses the optimal *δ*–*p*_O_2__ relationship for the entire *p*_O_2__ range relevant to CLWGS (or other similar applications) for a given temperature. Fig. 8 shows the *δ*–*p*_O_2__ relationship of three well-known non-stoichiometric oxides, all of which are far from the ideal case identified in this work. There are two possible ways to address this challenge.

First, the solid phase used for these reactions can be made of a combination of non-stoichiometric materials, so the overall composite curve approximates the optimal shape. Stoichiometric materials should generally be avoided as they introduce discontinuities in oxygen content at specific oxygen potentials. This approach is particularly useful at temperatures below 1093 K for CLWGS, where there is a window of operation bounded by the composite curves representing the H_2_O and CO streams, respectively (see Fig. 7). As long as the composite curve of the solid lies within this region, there is only a small penalty to the efficiency of the overall process, as evident from Table 1.

Second, instead of using a chemical looping scheme, the oxygen exchange between H_2_O and CO can also take place in a counter-current membrane reactor, akin to that proposed by Bulfin *et al.* for similar processes.^27,34^ In this case, only a single, oxygen conducting, non-stoichiometric solid is needed to facilitate the oxygen transport, and the solid acts as the “wall” of the oxygen exchanger and no longer plays a significant role in the pinch analysis. Therefore, its *δ*–*p*_O_2__ relationship no longer affects the efficiency of the process, so long as the oxygen transport rate is sufficiently fast across the full range of *p*_O_2__ required. However, the challenge lies in the cost-effective manufacturing of such a ceramic membrane reactor that remains well sealed at the operating temperature.

## 6. Conclusions

This work has highlighted the importance of matching the *δ*–*p*_O_2__ relationship between the gas phase and solid phase for CLWGS, and by extension, any chemical looping type heterogenous reactions that are thermodynamically limited. Another important factor to consider is the oxygen capacity available per unit mass of the OCM in the relevant range of oxygen chemical potentials. Based on these key characteristics, an analogy between CLWGS and heat exchanger networks is drawn, and the pinch analysis can be applied for chemical reactions between unmixed streams for their rational design and optimisation.

## Nomenclature

A: Cation site in the perovskite structure (ABO_3_)
B: Cation site in the perovskite structure (ABO_3_)
CLWGS: Chemical looping water-gas shift
*K*_ox_
: Equilibrium constant for eqn (4)
*K*_red_
: Equilibrium constant for eqn (5)
*L*
: Bed length (m)
*F*_g_
: Molar flowrate of gas phase (mol s^−1^)
*F*_O_
: Molar flowrate of oxygen in gas phase (mol s^−1^)
*N*_OCM_
: Moles of OCM in the packed bed reactor (mol)
OCM: Oxygen-carrier material
*T*
: Temperature (K)
WGS: Water-gas shift
*X*_CO_
: Conversion of CO to CO_2_
*X*_H_2_O_
: Conversion of H_2_O to H_2_
*k*_grad_
: Steepness of the logistic function
*p*_O_2__
: Oxygen partial pressure (bar)
*p*_O_2_mid_
: Oxygen partial pressure at the midpoint of the logistic function (bar)
*y*_*i*_
: Mole fraction of gaseous species *i*
*r*
: Radius of the packed bed (m)
*t*
: Half cycle duration (s)
*δ*
: Degree of oxygen non-stoichiometry of OCM
*δ*_ox_
: Degree of oxygen non-stoichiometry of OCM under the most oxidising operating condition
*δ*_red_
: Degree of oxygen non-stoichiometry of OCM under the most reducing operating condition
*δ*_max_
: Maximum degree of oxygen non-stoichiometry of OCM achievable
*δ*_min_
: Minimum degree of oxygen non-stoichiometry of OCM achievable
*δ*_n_
: Normalised degree of oxygen non-stoichiometry of OCM achievable
*ε*
: Voidage of the packed bed
*λ*_O_
: Dimensionless number defined by the ratio of solid phase oxygen capacity to gas phase oxygen capacity
*ρ*_solid_
: Molar density of the solid phase (mol m^−3^)
*ρ*_gas_
: Molar density of the gas phase (mol m^−3^)
Ω: Subscript representing equilibrium

## Author contributions

M. Selim Ungut: data curation, formal analysis, investigation, methodology, software, validation, visualisation, writing – original draft. Ian S. Metcalfe: funding acquisition, supervision, writing – review & editing. Wenting Hu: conceptualisation, funding acquisition, methodology, project administration, validation, writing – review & editing.

## Conflicts of interest

There are no conflicts to declare.

## Supplementary Material

RE-010-D4RE00454J-s001

RE-010-D4RE00454J-s002

## Data Availability

The MATLAB scripts used to generate results presented in this work can be found at https://doi.org/10.25405/data.ncl.27055339. The version of MATLAB employed for this study is version R2021b.
