# Supplementary material for: Identifying the ideal thermodynamics of non-stoichiometric oxygen-carrier materials for chemical looping water-gas shift
Source: React Chem Eng. 2025 Jan 15;10(4):800–9. doi: 10.1039/d4re00454j (PMC13559779; doi:10.1039/d4re00454j)
Supplement: Supplementary file 1 [file RE-010-D4RE00454J-s001.pdf]

This journal is © The Royal Society of Chemistry 2025

Matlab code for CLWGS reactor model

The function "ReactorModel.m" outputs a .mat file containing the gas concentrations and corresponding time resolution, cycle numbers and δ values.

"ReactorModel.m"
Initialise code and set thermodynamic properties

```
function ReactorModel
%WGS thermodynamic constants under isothermal conditions
T = 1093.15;                          % Reactor temperature (K)
R = 8.314;                               % Universal gas constant (J/molK)
Kox = 1.19323807516078E-09;           %Equilibrium constant for oxidation half cycle at 1093
K,  H2O = H2 + 1/2 O2
Kred = 1/1.19323807516078E-09;        %Equilibrium constant for reduction half cycle at 1093
K,  CO + 1/2 O2 = CO2

% Kox= 7.65449604394292E-08;  %Equilibrium constant for oxidation half cycle at 1293K
% Kred= 7542158.25100165;     %Equilibrium constant for reduction half cycle at 1293K

% Kox= 1.09706E-08;           %Equilibrium constant for oxidation half cycle at 1193K
% Kred= 67788067.7;           %Equilibrium constant for reduction half cycle at 1193K

% Kox= 7.08688E-11;           %Equilibrium constant for oxidation half cycle at 993K
% Kred= 20892750937;          %Equilibrium constant for reduction half cycle at 993K

% Kox=2.47252294976646E-12;   %Equilibrium constant for oxidation half cycle at 893K
% Kred=967456158223.589;      %Equilibrium constant for reduction half cycle at 893K
%Cycle settings

Trun = 60;                                % Length of half cycle in seconds
Number_of_cycles =25;                     % Number of redox cycles
%OCM δ-pO2 relationship

dmax=1;   % Maximum degree of non-stoichiometry
Midpoint=-17.8465457942801; % Log to the base ten of the midpoint of the logistic fit for the
δ-pO2 relationship
Kgrad = -1.151292546497; % Steepness of logistic function
```

OCM particle properties

```
rhoOCM=23071.57976; % Skeletal density of OCM at room temperature (mol/m3)
porosity=0; % Particle porosity, set to zero assuming unhindered gas flow through the bed
%Bed properties

L = 0.08;       % Length of bed (m)
dl=L/100;      % Discretised length
l=0:dl:L;       % Create uniform mesh, row vector
numInc=length(l)-1; % Number of individual increments used in simulation
r = 0.002;      % Bed radius (m)
A = pi()*r^2;   % Bed cross section (m2)
epsilon =0.8;                         % Voidage of bed
rhogas=1e5/R/T;                        % Gas phase density (mol/m3)
flowrate_ox=50/1000/24/60;      % Total gas flow during oxidation half cycle (mol/s)
flowrate_red=50/1000/24/60;     % Total gas flow during reduction half cycle (mol/s)
Vg=dl*A*epsilon*rhogas;                % Moles total gas in one increment
ms= rhoOCM*(1-porosity)*(1-epsilon)*A*dl;   % Moles of OCM per control volume
molsOCM=L*A*(1-epsilon)*rhoOCM*(1-porosity); % Total moles of OCM in bed
```

Inlet gas concentrations as mole fractions and oxygen partial pressure

```
y_total=0.05;    % Total concentrations of CO,CO2,H2,H2O must sum to 0.05 in argon
y_CO_inlet=0.04999999999;    % CO inlet
y_H2O_inlet=0.04999999999;   % H2O inlet
y_CO2_inlet=(y_total-y_CO_inlet);      % CO2 inlet
y_H2_inlet= (y_total-y_H2O_inlet);% H2 inlet

p_ox = Kox^2*((y_H2O_inlet-y_H2_inlet)/y_H2_inlet)^2;          % pO2 of inlet stream during
oxidation
p_red=(1/Kred)^2*(y_CO2_inlet/y_CO_inlet)^2;                   % pO2 of inlet stream during
reduction

delta_ox = dmax./(1+exp(-Kgrad.*(log10(p_ox)-Midpoint))); % δ value of inlet stream during
oxidation
delta_red = dmax./(1+exp(-Kgrad.*(log10(p_red)-Midpoint))); % δ value of inlet stream during
reduction
```

Calculating the value of λ_O for the simulation

```
lambda_O = ((delta_red-delta_ox)*molsOCM)/(flowrate_ox*Trun*0.04999999999); % Value of λO.
Insert a breakpoint here to confirm value when running simulation
```

Initialise parameters for ODE

```
IC=y_CO_inlet/(1+Kred*p_ox^0.5)*ones(numInc,1); % Initial condition for reduction half cycle

t=linspace(0,Trun,6000); % Set resolution for time

pCO=zeros(numInc,length(t),Number_of_cycles); % Predefine matrix sizes to speed up
calculations
pH2O=zeros(numInc,length(t),Number_of_cycles);
pO2=p_ox*ones(numInc,1);

J_red = spdiags([ones(numInc,1),ones(numInc,1)],[-1,0],numInc,numInc); % Set up jacobian
patterns
red_options=odeset('RelTol',1e-13,'AbsTol',1e-14, 'Stats', 'off',  'JPattern', J_red);

J_ox = spdiags([ones(numInc,1),ones(numInc,1)],[0,1],numInc,numInc);
ox_options=odeset('RelTol',1e-13,'AbsTol',1e-14, 'Stats', 'off',  'JPattern', J_ox);
```

ODE solving for each cycle

```
for cycle=1:Number_of_cycles

    sol_red = ode45(@red_ode,[0,Trun],IC,red_options); % Use ode15s if stiff
    pCO(:,:,cycle)=deval(sol_red,t); %Storing values of CO concentration from half cycle
    P_eq=(1/Kred)^2*((y_total-pCO(:,end,cycle))./pCO(:,end,cycle)).^2; %pO2 at each point in
the bed at end of half cycle
    IC=y_total./(1+Kox./P_eq.^0.5); %Initial condition for oxidation half cycle

    sol_ox=ode45(@ox_ode,[0,Trun],IC,ox_options); % Use ode15s if stiff
    pH2O(:,:,cycle)=deval(sol_ox,t); %Storing values of CO concentration from half cycle
    P_eq=(Kox)^2*(pH2O(:,end,cycle)./(y_total-pH2O(:,end,cycle))).^2; %pO2 at each point in
the bed at end of half cycle
    IC=y_total./(1+Kred*P_eq.^0.5); %Initial condition for reduction half cycle

end
%REDUCTION HALF CYCLE

function dydt=red_ode(t,u) %u represents the concentration of CO
pO2=(1/Kred)^2*((y_total-u)./u).^2;
```

```
dddp=(dmax*Kgrad.*exp(Kgrad*(log10(pO2)-Midpoint)))./(pO2*log(10).*(exp(Kgrad*(log10(pO2)-
Midpoint))+1).^2); %Derivative of logistic function
dpdy=(-2*(1/Kred)^2*y_total*(y_total-u))./(u.^3); % Finding dpO2/dyCO

%     Setting up continuity equations
dydt=zeros(numInc,1);
dydt(1)=flowrate_red.*(u(1)-y_CO_inlet)./(-dpdy(1).*dddp(1).*ms-Vg); % Material balance for
increment 1
    for j=2:numInc
        dydt(j)=flowrate_red.*(u(j)-u(j-1))./(-dpdy(j).*dddp(j).*ms-Vg); % Material balance
for increments 2 to numInc
    end
end
%OXIDATION HALF CYCLE

function dydt=ox_ode(t,v) %v represents the concentration of H2O
pO2=(Kox)^2*(v./(y_total-v)).^2;
dddp=(dmax*Kgrad.*exp(Kgrad*(log10(pO2)-Midpoint)))./(pO2*log(10).*(exp(Kgrad*(log10(pO2)-
Midpoint))+1).^2); %Derivative of logistic function
dpdy=(-2*(Kox)^2*y_total*v)./((v-y_total).^3); % Finding dpO2/dyH2O

%     Setting up continuity equations
dydt=zeros(numInc,1);
dydt(numInc)=flowrate_ox.*(v(numInc)-y_H2O_inlet)./(dpdy(numInc).*dddp(numInc).*ms-Vg); %
Material balance for increment numInc
    for j=1:numInc-1
           k=numInc-j;
        dydt(k)=flowrate_ox.*(v(k)-v(k+1))./(dpdy(k).*dddp(k).*ms-Vg); % Material balance for
increments numInc-1 to 1
    end
end
```

Saving the simulation data

```
pox=(Kox)^2*(pH2O./(y_total-pH2O)).^2;
lpox=log10(pox);

pred=(1/Kred)^2*((y_total-pCO)./pCO).^2;
lpred=log10(pred);

delta_ox=dmax./(1+exp(-Kgrad*(lpox-Midpoint)));
delta_red=dmax./(1+exp(-Kgrad*(lpred-Midpoint)));

fname=sprintf('CLWGS_Ideal_%d_cycles_%d_secs_%.0f_K.mat',Number_of_cycles,Trun,T);
save(fname,'pCO', 'pH2O', 'delta_ox', 'delta_red');
end
```

Matlab code to visualise output data from CLWGS reactor model

"DataView.m" can be used to quickly plot the data output from ReactorModel.m as concentration profiles and conversions per cycle. To use, load in the data output from ReactorModel.m and enter values for 'bedlength', 'Res' and 'cycles'. Also set the value of 'durationofsinglecycles' according to the feed duration that was used in the simulation.

"DataView.m"
Initialise code and load data

```
%%Script to quickly process model data
%
%Load data ouput from model

```

```
load CLWGS_Ideal_25_cycles_60_secs_1093_K.mat % Placeholder only. Enter the name of your own
.mat file here.
%Values to input

bedlength=100; % Enter the number of individual increments that were used in simulation
Res=6000; % Enter resolution of time dimension used in simulation
cycles=25; % Enter the number of cycles used in simulation

durationofsinglecycle=(Res/60)*4; % Use (Res/60)*20 when using 5 min feed durations
                                  % Use (Res/60)*16 when using 4 min feed durations
                                  % Use (Res/60)*12 when using 3 min feed durations
                                  % Use (Res/60)*8 when using 2 min feed durations
                                  % Use (Res/60)*4 when using 1 min feed durations
```

Preparing gas outlet data

```
pH2=0.05-pH2O; % Calculating concentration of H2. Concentration of H2 and H2O must sum to 0.05
in argon
pCO2=0.05-pCO; % Calculating concentration of CO2. Concentration of CO2 and CO must sum to
0.05 in argon
j=0; % Setting for argon hold duration
for i=1:cycles
H2((1+(j*Res)):((1+(j*Res))+(Res-1)))=pH2(1,:,i);
H2O((1+(j*Res)):((1+(j*Res))+(Res-1)))=pH2O(1,:,i);
CO2((1+(j*Res)):((1+(j*Res))+(Res-1)))=0;
CO((1+(j*Res)):((1+(j*Res))+(Res-1)))=0;
j=j+2; % How long the argon hold between half cycles is
H2((1+(j*Res)):((1+(j*Res))+(Res-1)))=0;
H2O((1+(j*Res)):((1+(j*Res))+(Res-1)))=0;
CO2((1+(j*Res)):((1+(j*Res))+(Res-1)))=pCO2(bedlength,:,i);
CO((1+(j*Res)):((1+(j*Res))+(Res-1)))=pCO(bedlength,:,i);
j=j+2; % How long the argon hold between half cycles is
end
clear j
```

Plot concentration profile

```
time=(1:(length(H2)))/60;
plot (time,H2,time,CO2,time,H2O,time,CO);
legend('H2','CO2','H2O','CO')
xlabel('Time (mins)') % x-axis label
ylabel('Conc.') % y-axis label
```

Preparing data for integration

```
time=(1:(length(H2)))/60;
ConcCO=[CO',ones(length(CO'),1)];
ConcCO2=[CO2',ones(length(CO2'),1)];
ConcH2=[H2',ones(length(H2'),1)];
ConcH2O=[H2O',ones(length(H2O'),1)];
time_trans=time';
totalhalfcycles=0; %at start
```

Integration loop (using trapz function)

```
for j=(cycles-10):cycles % Calculates conversions for specified range of cycles. Use a smaller
range for faster computation time.
        realstarttime=(0)+(durationofsinglecycle*(j-1));
```

```
        realendtime=(durationofsinglecycle-
(durationofsinglecycle/4))+(durationofsinglecycle*(j-1));
        range_data=[realstarttime 0 ; realendtime 0];
        range_index1 = knnsearch(time_trans,range_data(1,1));
        range_index2 = knnsearch(time_trans,range_data(2,1));
        totalrange=range_index2-range_index1+1;

        COforintegrate=zeros(totalrange,1);
        CO2forintegrate=zeros(totalrange,1);
        H2forintegrate=zeros(totalrange,1);
        H2Oforintegrate=zeros(totalrange,1);
        timeforintegrate=zeros(totalrange,1);

        for k=1:totalrange
            H2Oforintegrate(k,1)=ConcH2O(k+range_index1-1,1);
            H2forintegrate(k,1)=ConcH2(k+range_index1-1,1);
            CO2forintegrate(k,1)=ConcCO2(k+range_index1-1,1);
            COforintegrate(k,1)=ConcCO(k+range_index1-1,1);
            timeforintegrate(k,1)=time_trans(k+range_index1-1,1);
        end

       IntegralCO(j+totalhalfcycles,1)=trapz(timeforintegrate,COforintegrate);
       IntegralCO2(j+totalhalfcycles,1)=trapz(timeforintegrate,CO2forintegrate);
       IntegralH2(j+totalhalfcycles,1)=trapz(timeforintegrate,H2forintegrate);
       IntegralH2O(j+totalhalfcycles,1)=trapz(timeforintegrate,H2Oforintegrate);

       clearvars totalrange timeforintegrate COforfintegrate CO2forintegrate H2forintegrate
H2Oforintegrate
end
```

Plot conversions

```
H2conversion=IntegralH2./(IntegralH2+IntegralH2O);
CO2conversion=IntegralCO2./(IntegralCO2+IntegralCO);
nostp=length(IntegralH2); %number of cycles in plot
cycle(:,1) = linspace(1,nostp,nostp);

h0 = figure('units','normalized','outerposition',[0 0 1 1]);
plot(cycle, CO2conversion(:,1),'k-.',cycle,H2conversion(:,1),'k--');
legend('CO2','H2')
xlabel('Cycle Number') % x-axis label
ylabel('Conversion mol/mol') % y-axis label
```
