# Supplementary material for: Identifying the ideal thermodynamics of non-stoichiometric oxygen-carrier materials for chemical looping water-gas shift
Source: React Chem Eng. 2025 Jan 15;10(4):800–9. doi: 10.1039/d4re00454j (PMC13559779; doi:10.1039/d4re00454j)
Supplement: Supplementary file 2 [file RE-010-D4RE00454J-s002.pdf]

This journal is © The Royal Society of Chemistry 2025

Supplementary information for:

Identifying the ideal thermodynamics of non-stoichiometric oxygen-carrier materials for chemical looping water-gas shift

Derivation of dimensionless number λ_O

The dimensionless number λ_O, which is used extensively throughout the paper, is defined by the ratio of moles of usable oxygen in the solid phase $N_{OCM,\,oxygen,\,usable}$, divided by the moles of oxygen present in the passing gas phase stream during a half cycle $N_{Gas,\,oxygen}$.

$$\lambda_O = \frac{N_{OCM,\,oxygen,\,usable}}{N_{Gas,\,oxygen}} \quad \text{Eq. S1}$$

The moles of oxygen present in the passing gas phase stream in a half cycle is given by the following,

$$N_{Gas,\,oxygen} = F_O t \quad \text{Eq. S2}$$

where F_O refers to the molar flowrate of oxygen in the gas phase and t refers to the duration of the half cycle.

The usable moles of oxygen in the OCM is logically given by the following,

$$N_{OCM,\,oxygen,\,usable} = N_{OCM,\,oxygen,\,oxidation} - N_{OCM,\,oxygen,reduction} \quad \text{Eq. S3}$$

where $N_{OCM,\,oxygen,\,oxidation}$ and $N_{OCM,\,oxygen,\,reduction}$ refer to the moles of oxygen in the OCM at the end of the oxidation and reduction half cycles respectively.

The moles of oxygen in the OCM at the end of the oxidation and reduction half cycles are given by,

$$N_{OCM,\,oxygen,\,oxidation} = (S - \delta_{ox})N_{OCM} \quad \text{Eq. S4}$$

$$N_{OCM,\,oxygen,reduction} = (S - \delta_{red})N_{OCM} \quad \text{Eq. S5}$$

where N_{OCM} refers to the moles of OCM, S refers to the oxygen stoichiometry when δ is equal to 0 (e.g. for the OCM $La_{0.6}Sr_{0.4}FeO_{3-\delta}$ the value of S is equal to 3), and δ_{red} and δ_{ox} are

the degrees of non-stoichiometry of the OCM during steady cycling at the end of the reduction and oxidation half cycles respectively.

Subtracting Eq. S4 from Eq. S5 leads to the following equation:

$$N_{OCM,\,oxygen,\,usable} = (\delta_{red} - \delta_{ox})N_{OCM} \qquad \text{Eq. S6}$$

Substituting Eq. S2 and Eq. S6 into Eq. S1 therefore leads to the following equation for the dimensionless number λ_O:

$$\lambda_O = \frac{(\delta_{red} - \delta_{ox})N_{OCM}}{F_O t} \qquad \text{Eq. S7}$$

Verifying λ_O using different values of $\delta_{red} - \delta_{ox}$, t, N_{OCM} and F_O

The simulation results in Table S1 demonstrate that the conversions are dependant only on the values of $\log_{10} p_{O_2 mid}$, k_{grad} and λ_O. For example, a λ_O value of 0.5 gives X_{CO} and X_{H_2O} values of ~50% regardless of how λ_O is derived with changes to $\delta_{red} - \delta_{ox}$ and N_{OCM}.

Table S1: Conversions determined from the simulation using different values of λ_O using various $\delta_{red} - \delta_{ox}$, F_{O_2}, t and N_{OCM} values. The values of k_{grad} and $\log_{10} p_{O_2 mid}$ are kept constant at -1.15 and -17.8 respectively. The values of conversion that are shown are those during steady cycling when the difference between X_{CO} and X_{H_2O} is < 0.01.

λ_O	$\delta_{red} - \delta_{ox}$	F_O (mol min^{-1})	t (s)	N_{OCM} (mols)	X_{H_2O}, X_{CO}
1	0.0225	1.7361×10^{-6}	60	0.004639	0.94
1	0.0225	8.6806×10^{-7}	120	0.004639	0.94
1	0.0225	1.7361×10^{-6}	120	0.009278	0.94
1	0.0449	3.4722×10^{-6}	60	0.004639	0.94
0.5	0.0112	1.7361×10^{-6}	60	0.004639	0.50
0.5	0.0112	8.6806×10^{-7}	120	0.004639	0.50
0.5	0.0112	1.7361×10^{-6}	120	0.009278	0.50
0.5	0.0225	3.4722×10^{-6}	60	0.004639	0.50

Fig. S1a and Fig. S1d show that there are no noticeable changes to the shapes of the outlet concentration profiles at λ_O = 1 when using different F_O and $\delta_{red} - \delta_{ox}$ values. Meanwhile the outlet concentration profile at 60s feed durations compared to 120 s feed durations in

Fig. S1a and Fig. S1d compared to Fig. S1b and Fig. S1c is a half-scaled version of the 120 s profile along the x-axis. This indicates that as expected while a greater volume of H_2 is

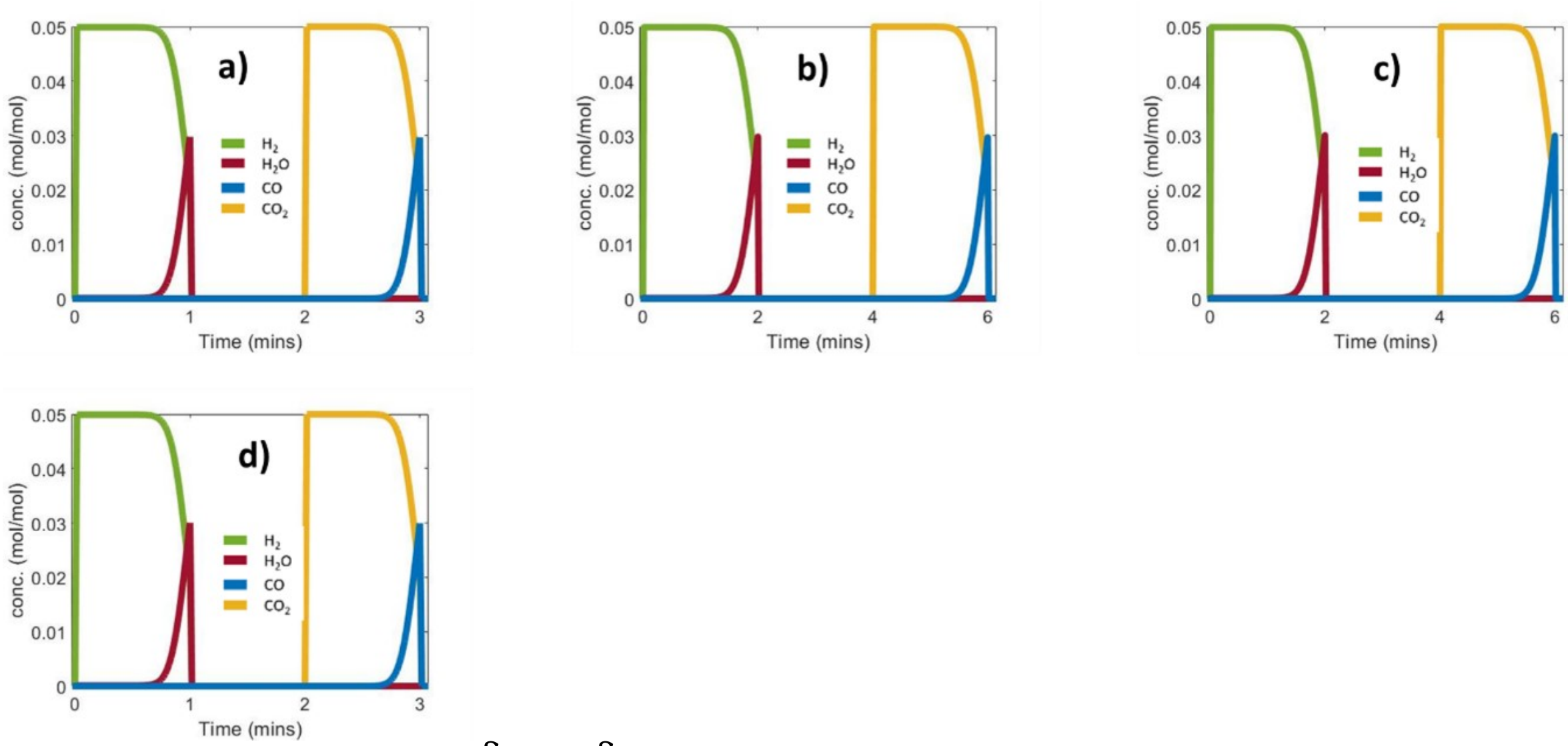

possible when the value of $\delta_{red} - \delta_{ox}$ is greater or more moles of OCM are used, the conversions and outlet product quality is still determined by the value of λ_O and the specific $\log_{10} p_{O_{2}mid}$ and k_{grad} of the δ-p_{O_2}curve.

Figure S1: Comparison on the effect on outlet concentration profiles for λ_O = 1 when using a) $\delta_{red} - \delta_{ox}$ = 0.0225, F_O = 1.7361 × 10^{-6} mol min^{-1}, t = 60s, N_{OCM} = 0.004639 mols, b) $\delta_{red} - \delta_{ox}$ = 0.0225, F_O = 8.6806 × 10^{-7} mol min^{-1}, t = 120s, N_{OCM} = 0.004639 mols, c) $\delta_{red} - \delta_{ox}$ = 0.0225, F_O = 1.7361 × 10^{-6} mol min^{-1}, t = 120s, N_{OCM} = 0.009278 mols and d) $\delta_{red} - \delta_{ox}$ = 0.0449, F_O = 3.4722 × 10^{-6} mol min^{-1}, t = 60s, N_{OCM} = 0.004639 mols. The values of k_{grad} and $\log_{10} p_{O_{2}mid}$ are kept constant at -1.15 and -17.8 respectively. The profiles are taken

Fig. S2 shows the outlet concentration profiles corresponding to selected λ_O values for the optimal δ-p_{O_2} relationship. As expected, $\lambda_O < 1$ results in less available oxygen capacity and gives a decrease in conversions and product quality. For $\lambda_O <= 0.1$ significant quantities of unreacted feed gas start to be seen in the outlet concentration profiles. At $\lambda_O = 1 \times 10^{-3}$ it is seen that the inlet streams pass through the reactor almost unreacted with unacceptable product quality, while at a λ_O value of 1000 almost pure product H_2 and CO_2 can be seen

only limited by the p_{O_2} of the inlet feeds. It would still be possible to achieve high conversions at low λ_O values as the gradient of the curve k_{grad} and $\log_{10} p_{O_2 mid}$ are not changed, however a greater mass of OCM would need to be used to be to be able to do so which would be more expensive industrially. Alternatively an OCM with a similar δ-p_{O_2} relationship but with a larger maximum degree of oxygen non-stoichiometry could be used if it existed.

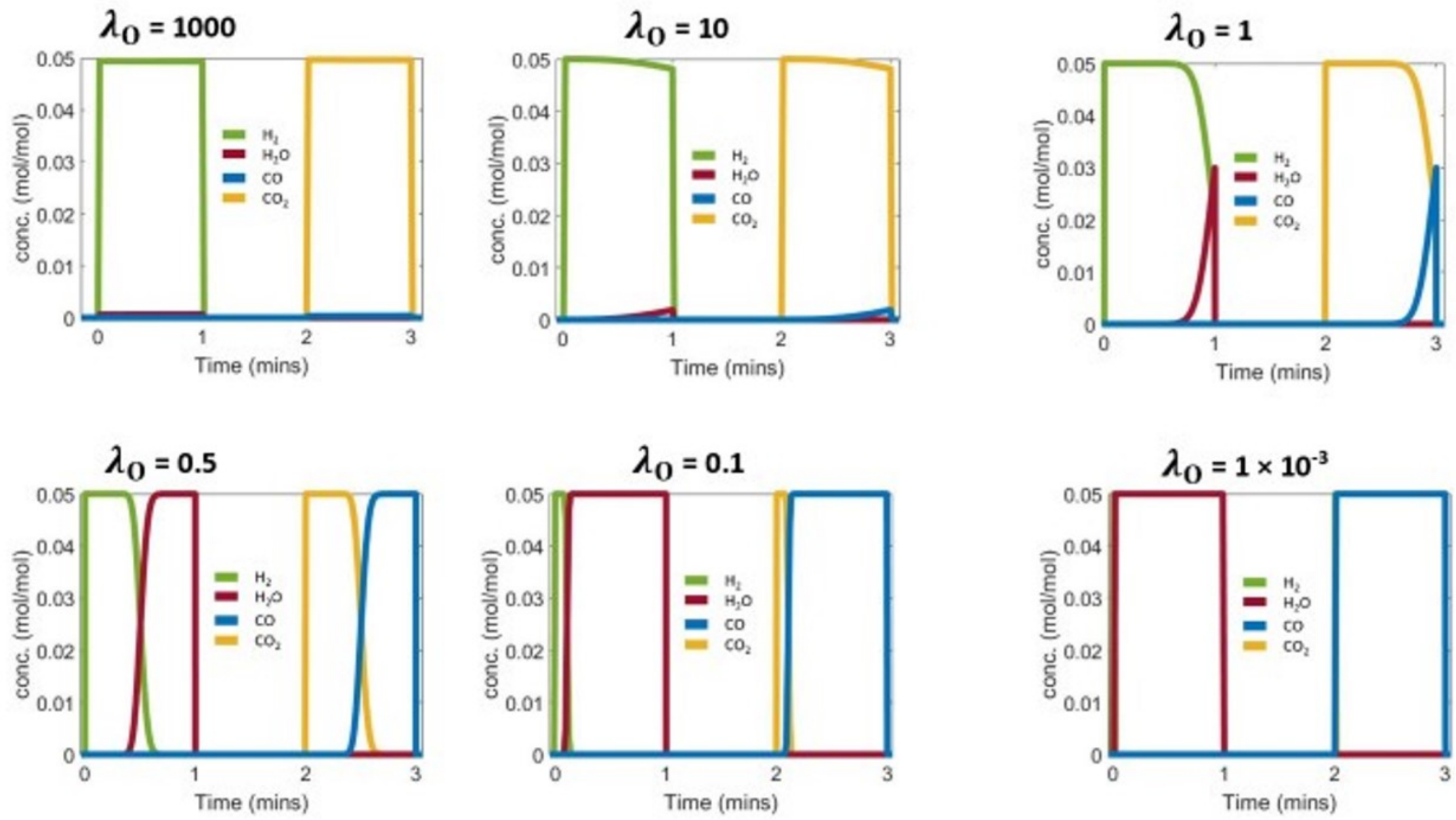

Figure S2: Outlet concentration profiles determined from the simulation using different values for λ_O of the δ-p_{O_2} plot at 1093 K. The values of k_{grad} and $\log_{10} p_{O_2 mid}$ are kept constant at -1.15 and -17.8 respectively. The profiles are taken during steady cycling when the difference between X_{CO} and X_{H_2O} is < 0.01.
